# Accelerated resolution of inflammation underlies sex differences in inflammatory responses in humans

**DOI:** 10.1172/JCI89429

**Published:** 2016-11-28

**Authors:** Krishnaraj S. Rathod, Vikas Kapil, Shanti Velmurugan, Rayomand S. Khambata, Umme Siddique, Saima Khan, Sven Van Eijl, Lorna C. Gee, Jascharanpreet Bansal, Kavi Pitrola, Christopher Shaw, Fulvio D’Acquisto, Romain A. Colas, Federica Marelli-Berg, Jesmond Dalli, Amrita Ahluwalia

**Affiliations:** William Harvey Research Institute, Barts and The London Medical School, Queen Mary University of London, London, United Kingdom.

## Abstract

**BACKGROUND.** Cardiovascular disease occurs at lower incidence in premenopausal females compared with age-matched males. This variation may be linked to sex differences in inflammation. We prospectively investigated whether inflammation and components of the inflammatory response are altered in females compared with males.

**METHODS.** We performed 2 clinical studies in healthy volunteers. In 12 men and 12 women, we assessed systemic inflammatory markers and vascular function using brachial artery flow-mediated dilation (FMD). In a further 8 volunteers of each sex, we assessed FMD response to glyceryl trinitrate (GTN) at baseline and at 8 hours and 32 hours after typhoid vaccine. In a separate study in 16 men and 16 women, we measured inflammatory exudate mediators and cellular recruitment in cantharidin-induced skin blisters at 24 and 72 hours.

**RESULTS.** Typhoid vaccine induced mild systemic inflammation at 8 hours, reflected by increased white cell count in both sexes. Although neutrophil numbers at baseline and 8 hours were greater in females, the neutrophils were less activated. Systemic inflammation caused a decrease in FMD in males, but an increase in females, at 8 hours. In contrast, GTN response was not altered in either sex after vaccine. At 24 hours, cantharidin formed blisters of similar volume in both sexes; however, at 72 hours, blisters had only resolved in females. Monocyte and leukocyte counts were reduced, and the activation state of all major leukocytes was lower, in blisters of females. This was associated with enhanced levels of the resolving lipids, particularly D-resolvin.

**CONCLUSIONS.** Our findings suggest that female sex protects against systemic inflammation-induced endothelial dysfunction. This effect is likely due to accelerated resolution of inflammation compared with males, specifically via neutrophils, mediated by an elevation of the D-resolvin pathway.

**TRIAL REGISTRATION.** ClinicalTrials.gov NCT01582321 and NRES: City Road and Hampstead Ethics Committee: 11/LO/2038.

**FUNDING.** The authors were funded by multiple sources, including the National Institute for Health Research, the British Heart Foundation, and the European Research Council.

## Introduction

There is substantial literature supporting the view that the lower incidence of cardiovascular disease (CVD) in premenopausal women compared with age-matched male counterparts and postmenopausal women (1, 2) suggests that female sex underlies a protective effect on the cardiovascular system (for reviews, see refs. 3, 4) — an effect that has been linked to an action of ovarian hormones, predominantly estrogens (for reviews, see refs. 5, 6). However, the exact target and molecular pathways that underlie the beneficial effects of female sex in humans has remained uncertain.

Most chronic CVD is characterized by endothelial dysfunction thought to be a consequence of an underlying systemic inflammation manifested as many of the symptoms associated with abnormal vascular regulation, including blood pressure dysregulation, inadequate tissue perfusion, capillary edema, and inflammatory cell recruitment. These changes ultimately lead to disease, including hypertension and atherosclerosis. Indeed, strong correlations between systemic markers of inflammation and inflammation of the arteries with increased risk for cardiovascular events have been demonstrated (7, 8). Likewise, endothelium dysfunction and event risk, disease prognosis, and disease severity are strongly associated (9–11). Importantly, experimental models in humans have confirmed the link between inflammation and endothelial dysfunction (12). There is also preclinical evidence suggesting that reduced systemic inflammation associated with preserved endothelium function in females is associated with protection against chronic CVD, particularly in models of atherosclerosis (for review, see ref. 13). Moreover, there is evidence predominantly from preclinical assessment that sex influences immune responses particularly with respect to the consequence of infection (14)

In accord with the above suggestions, using intravital microscopy we recently observed that leukocyte recruitment in response to cytokine IL-1β or TNF-α is reduced in female mice compared with age-matched male mice (8). This effect likely reflects an overall decrease in reactivity of the microvasculature in females, since the responses to two distinct proinflammatory cytokines were similarly suppressed. In addition, these findings are in agreement with studies from a range of animal models of inflammation, suggesting the existence of sex hormone–dependent male/female differences in inflammatory cell recruitment (15–19). However, whether similar pathways govern reduced inflammatory responses in humans is unknown and an issue we have aimed to address herein.

**Figure 1:**
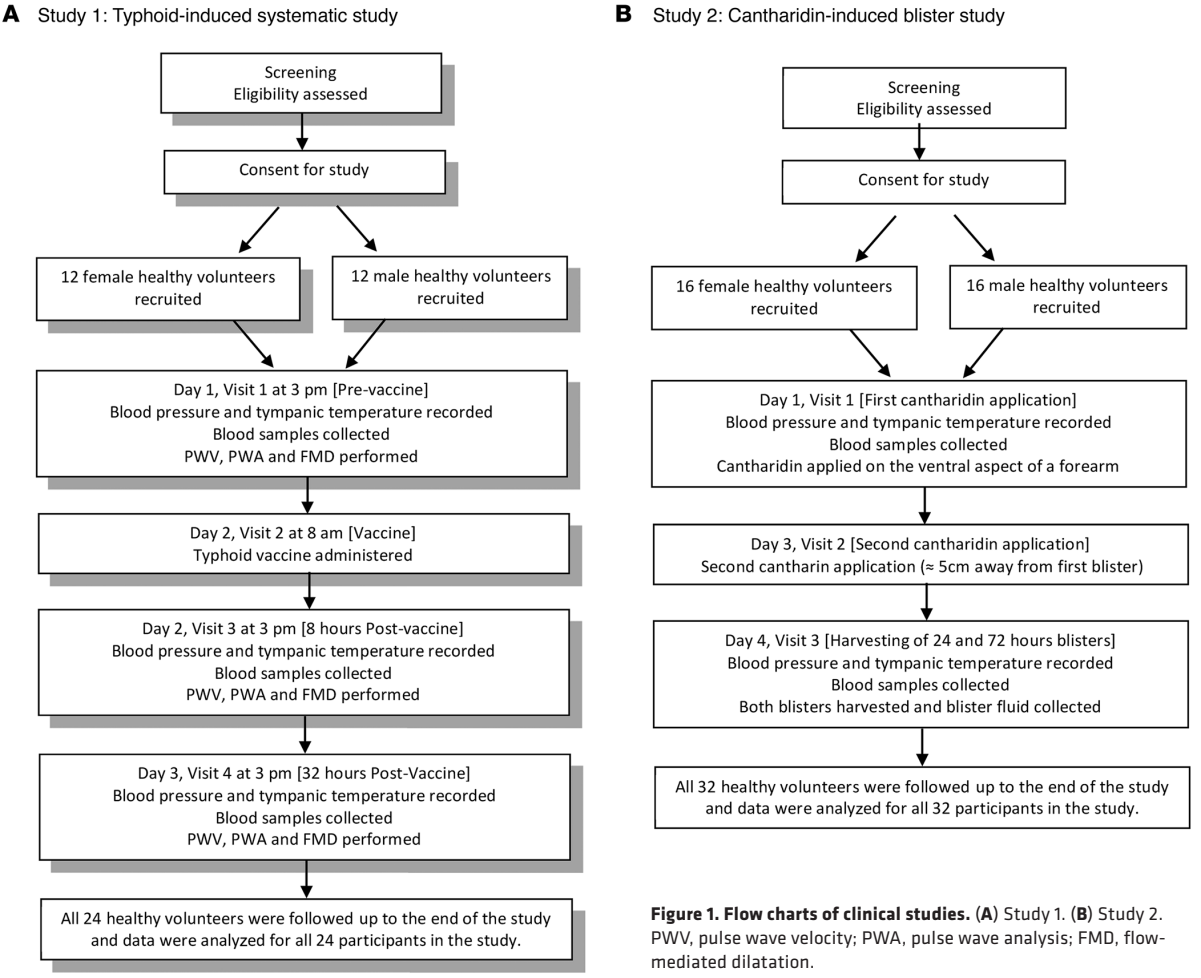
Flow charts of clinical studies. (**A**) Study 1. (**B**) Study 2. PWV, pulse wave velocity; PWA, pulse wave analysis; FMD, flow-mediated dilatation.

To test whether sex differences in inflammatory responses and pathways might underlie preserved endothelial function in females, we assessed both vascular and inflammatory responses in the systemic and local inflammation using the validated models of typhoid vaccine–induced endothelial dysfunction (12) (study 1) and cantharidin-induced blister formation (20) (study 2) in male and female healthy volunteers (Figure 1).

## Results

Baseline data for study 1 and 2 demonstrated that the volunteers were well matched in terms of age. Expected differences were observed in BMI, systolic blood pressure (SBP), and diastolic blood pressure (DBP) (Table 1).

### Typhoid vaccine induces a low-grade systemic inflammation in both males and females.

**Figure 2:**
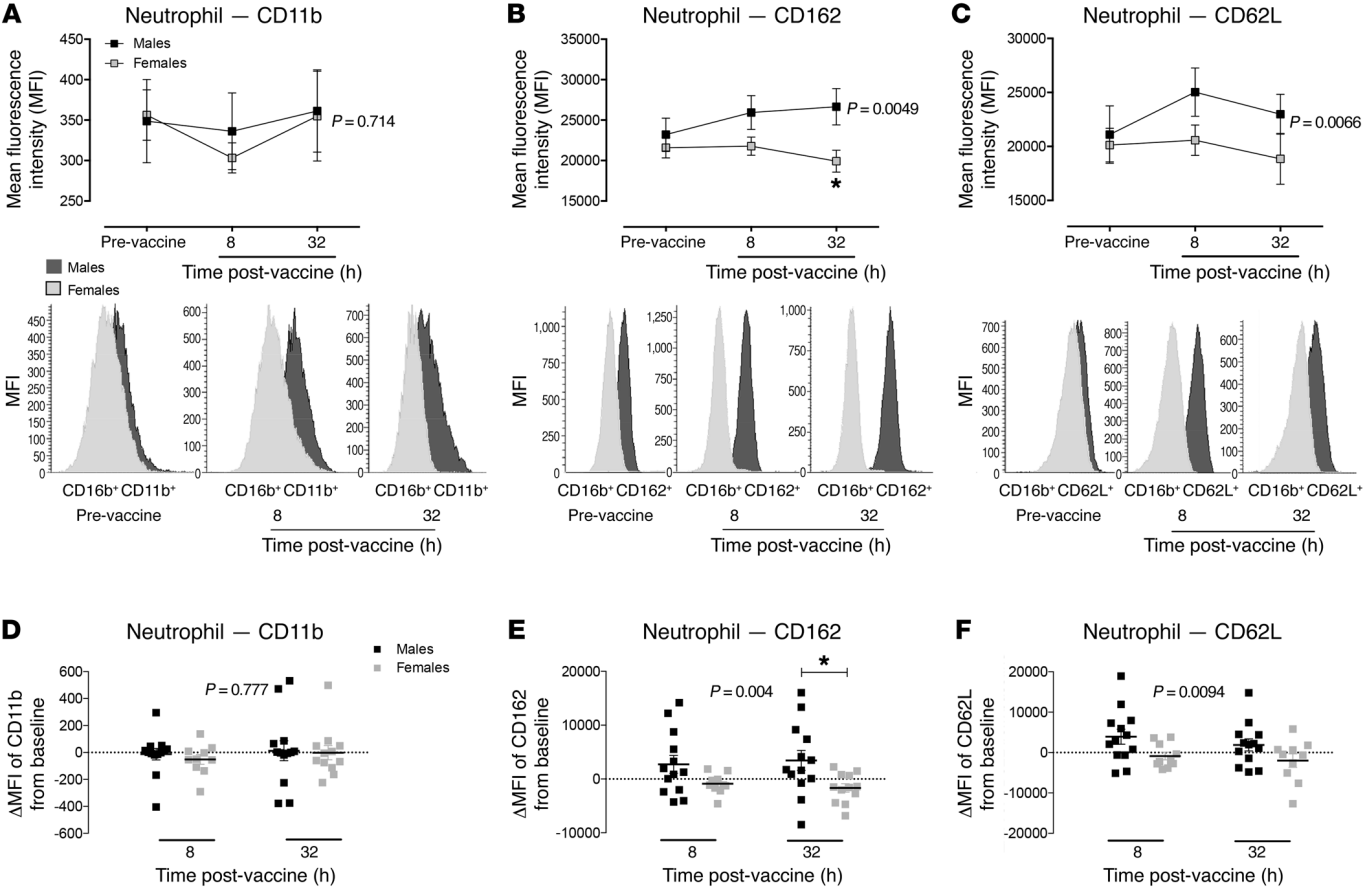
Low-grade systemic inflammation induced by typhoid vaccination enhances neutrophil activation state in healthy male but not female volunteers. Changes in expression of (**A**) CD11b, (**B**) CD162, and (**C**) CD62L on neutrophils in male and female healthy volunteers at baseline, 8 hours, and 32 hours following typhoid vaccine administration. Changes in expression of (**D**) CD11b, (**E**) CD162, and (**F**) CD62L from baseline in male and female healthy volunteers at 8 hours and 32 hours following typhoid vaccine administration. Data expressed as mean ± SEM of *n* = 12 male and female volunteers for all the panels. Statistical significance determined using 2-way ANOVA with Šídák’s post hoc analysis; *P < 0.05 for all panels.

Typhoid vaccination resulted in a rise in circulating leukocyte numbers in both sexes due almost entirely to a neutrophilia (Table 2), as per previous reports (12). Interestingly, it is notable that at baseline the numbers of wbc trended to higher values in females versus males. The differential counts demonstrate that this difference is due to greater numbers of neutrophils in females (*P* < 0.05); all other circulating cell numbers at baseline were similar between the sexes. Use of flow cytometry, to assess the relative activation state of the circulating cells, demonstrated that at baseline the expression of key adhesion molecules involved in inflammatory cell recruitment (CD11b, CD162, and CD62L) was largely similar between the sexes, except for an elevation in expression of activation markers on circulating monocytes in females (Supplemental Figure 1; supplemental material available online with this article; doi:10.1172/JCI89429DS1). Following typhoid vaccine, the activation state of circulating neutrophils relative to baseline was enhanced in males but not females, specifically with elevations of CD162 and CD62L (Figure 2), both cellular adhesion molecules that play a pivotal role in cell homing, rolling, and adhesion in an inflammatory scenario (21, 22) — an effect only evident on neutrophils and not on other cell types (Supplemental Figure 1). There were no measurable changes from baseline in platelet reactivity in either sex and at any of the time points tested (Supplemental Table 1). Circulating levels of C-reactive protein (CRP) and body temperature were not altered by typhoid vaccine (Table 2). To determine whether differences in the systemic inflammatory response might relate to differences in vaccine administration between the sexes, we assessed typhoid (IgM) antibody titers at 32 hours following vaccination. Our data showed no statistical differences in the antibody response (male: 10.4 ± 1.8 U/ml, *n* = 11 and female: 11.4 6 ± 1.6 U/ml, *n* = 10), suggesting that the above differences in systemic inflammation relate to biological differences between the sexes in the response to an inflammatory stress.

### Sex differences in the vascular response to typhoid vaccination.

**Figure 3:**
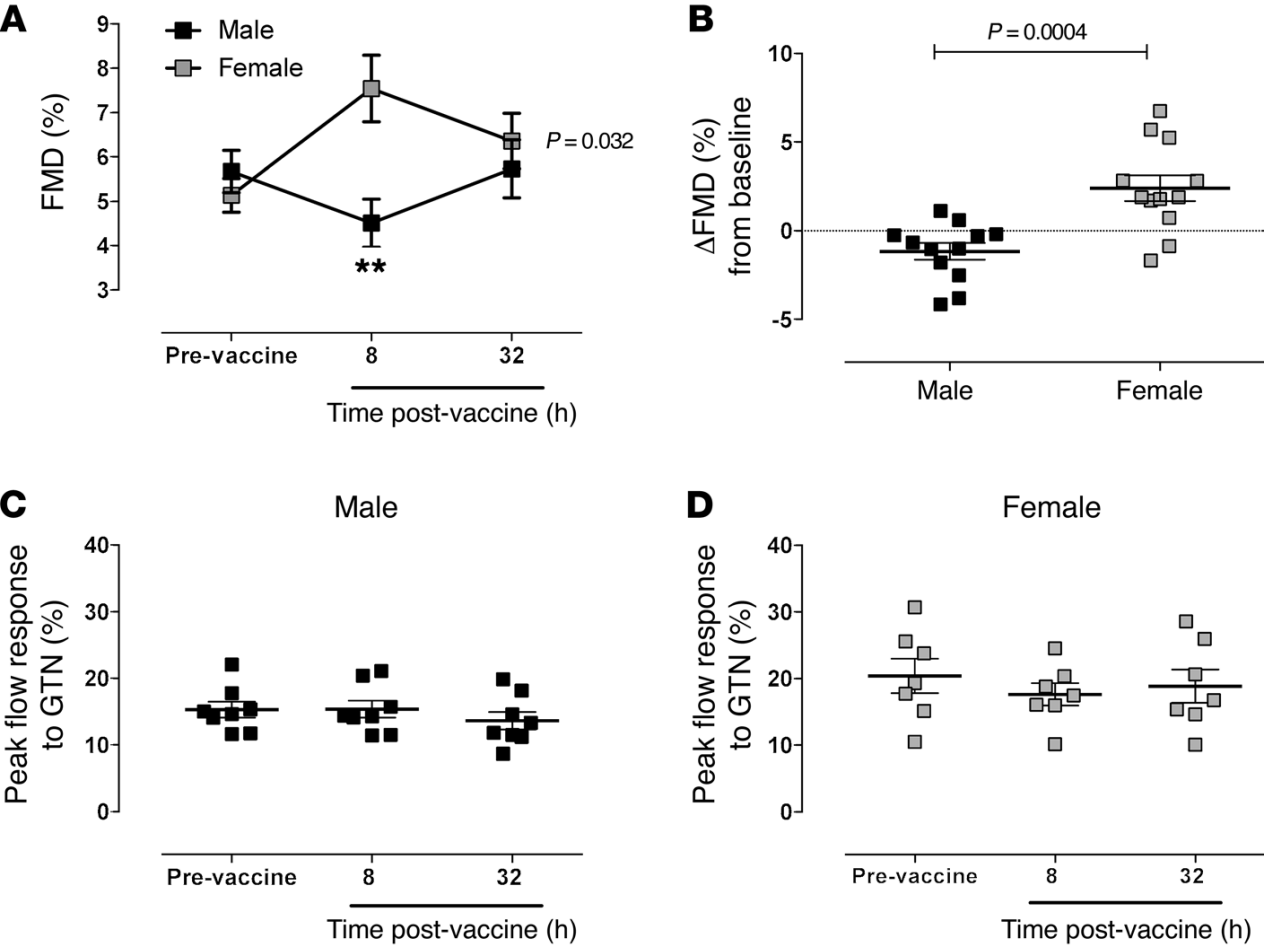
Low-grade systemic inflammation induced by typhoid vaccination does not cause endothelial dysfunction in healthy female volunteers. The effect of typhoid vaccination on (**A**) FMD measured at baseline and 8 and 32 hours following typhoid vaccination. (**B**)The effect of sex on the change in FMD at 8 hours following typhoid vaccination from baseline All data are expressed as mean ± SEM of *n* = 12 for each sex. Statistical significance determined using (**A**) 2-way ANOVA with Šídák’s post hoc analysis, **P < 0.01; and (**B**) Student’s 2-tailed unpaired test. Maximal dilatation of the brachial artery in response glyceryl trinitrate in (**C**) male and (**D**) female healthy volunteers measured at baseline and 8 and 32 hours following typhoid vaccination. Data expressed as mean ± SEM of *n* = 8. Statistical significance determined using 1-way ANOVA with Šídák’s post-hoc analysis.

Baseline ultrasound flow-mediated dilatation (FMD) responses were similar between the sexes; however, while typhoid vaccine tended to decrease the vascular response in males, in females the FMD response increased. This profile of FMD response was significantly different between the sexes, with the greatest difference evident at the 8-hour time point (*P* < 0.01; Figure 3, A and B). These changes were evident despite no change over time in baseline diameter or in the time taken to reach peak diameter following release of the cuff (Supplemental Table 2). Evidence suggests that the FMD response is due predominantly to triggering of endothelium-dependent NO generation. To assess whether this might underlie differences in the sexes, we measured nitrite and nitrate (NOx) levels. We found no significant differences in circulating NOx levels between the time points; however, interestingly post hoc correlation analyses demonstrated that circulating nitrite concentrations correlate directly with the magnitude of the FMD response in females but not males (Supplemental Figure 2). In contrast to FMD, typhoid vaccination did not alter the vasodilation response to sublingual GTN in either sex (Figure 3, C and D) and did not influence pulse wave velocity (PWV) or augmentation index through pulse wave analysis (PWA). Since the PWV and PWA are measures of arterial compliance determined predominantly by smooth muscle function (Supplemental Table 2), the data collectively suggest that the differences seen in the FMD response to typhoid vaccination were not due to underlying changes in smooth muscle reactivity.

### Sex differences in cantharidin-induced inflammation.

To explore more closely whether differences in inflammatory cells between males and females observed with typhoid vaccination might reflect sex-based differences in acute inflammatory responses, we used a validated cantharidin-based model of inflammation (20, 23). We chose this model since there is no noninvasive method in vivo to explore organ-specific cell and humoral components; therefore, we moved to an easily accessible organ-based model using skin inflammation. Cantharidin application at the time points assessed was not associated with a systemic inflammatory response in either sex, as reflected by the lack of change from baseline in peripheral blood markers of systemic inflammation, such as CRP, leukocyte count, and leukocyte differential (Supplemental Table 3). All volunteers reported that the typhoid vaccine caused minimal discomfort beyond the injection and that the cantharidin blisters were pain free other than an itching and/or tingling sensation a few hours after cantharidin application as the blisters formed. These observations are in line with other published studies (20, 24, 25), providing evidence that this is indeed a safe, reliable, and reproducible technique to study acute inflammation.

**Figure 4:**
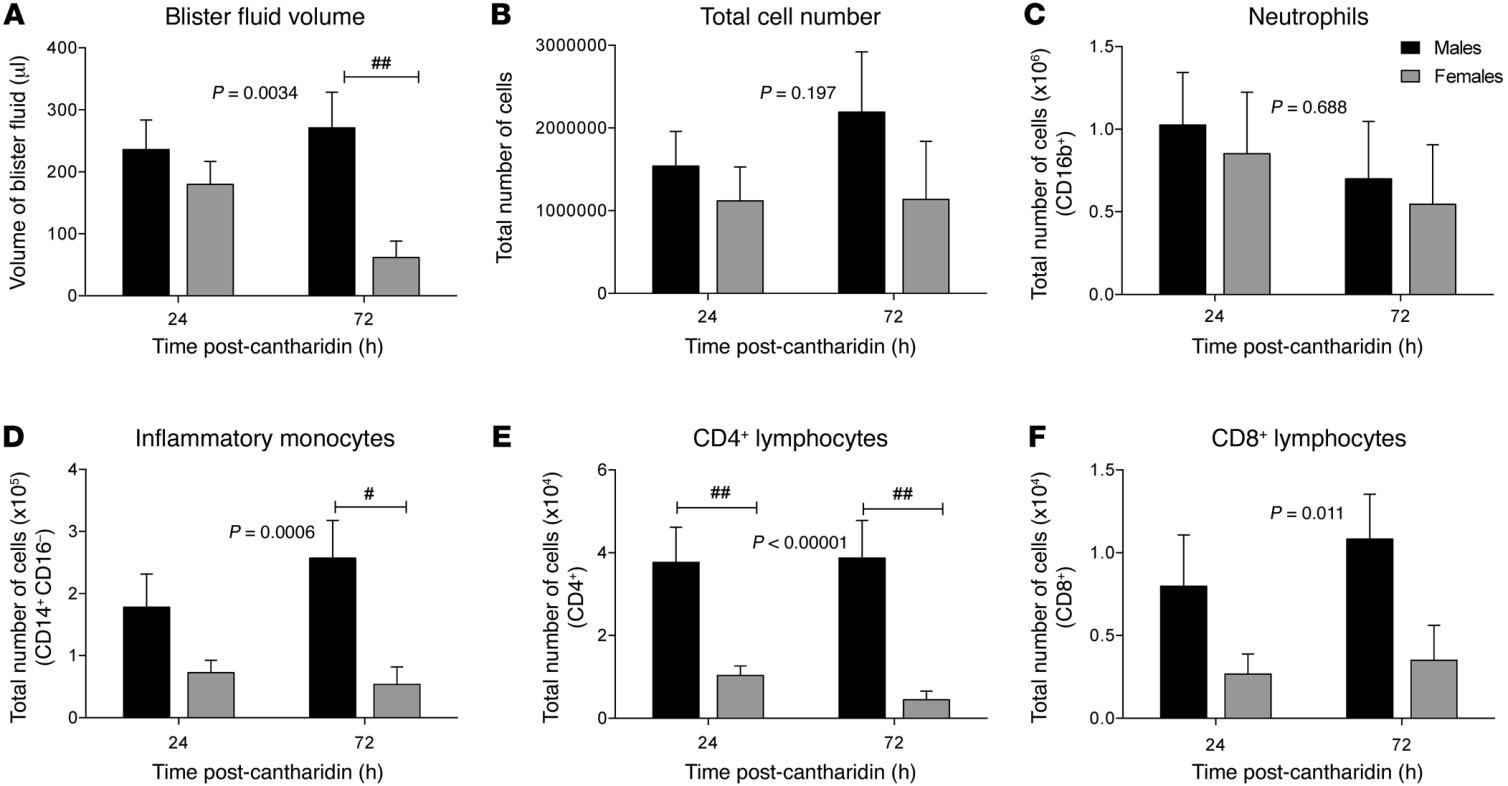
Reduced inflammatory response to cantharidin in female compared with male healthy volunteers. The total (**A**) volume of fluid, (**B**) cell count, (**C**) neutrophil count, (**D**) inflammatory monocyte count, and (**E**) CD4^+^ and (**F**) CD8^+^ T cell count at 24 hours and 72 hours following application of cantharidin to the volar aspect of the forearm. Data is shown as mean ± SEM of *n* = 16 of each sex for all the panels. Statistical analysis was determined using 2-way ANOVA followed by Šídák’s post tests; ^#^*P* < 0.05, ^##^*P* < 0.01 for all panels.

Within the cantharidin-induced blister, we found significantly lower edema in females compared with males (Figure 4A). Indeed, at the 72-hour time point, blisters had completely resolved in 11 females (70%) in comparison to only 5 males (using a 4 by 2 contingency table, the χ^2^ test demonstrated a significant difference between the sexes; *P* < 0.0019). While there was no significant difference (albeit a trend) in the total number of cells or in the number of neutrophils recruited into the blisters between the sexes (Figure 4, B and C), assessment of levels of other key inflammatory cell types and cell activation state exposed a prominent difference between the sexes (see Supplemental Figure 3 for typical scatter plots). In females the proportions and total numbers of inflammatory monocytes were significantly reduced compared with blisters from male volunteers, particularly at 72 hours (Figure 3D). Of note, there were also significantly lower levels of CD4^+^ and CD8^+^ T cells in females when compared with males at both the 24- and 72-hour time points. There were no differences in the numbers of either the intermediate monocyte or the resident monocyte subtype (see Supplemental Figures 4 and 5).

**Figure 5:**
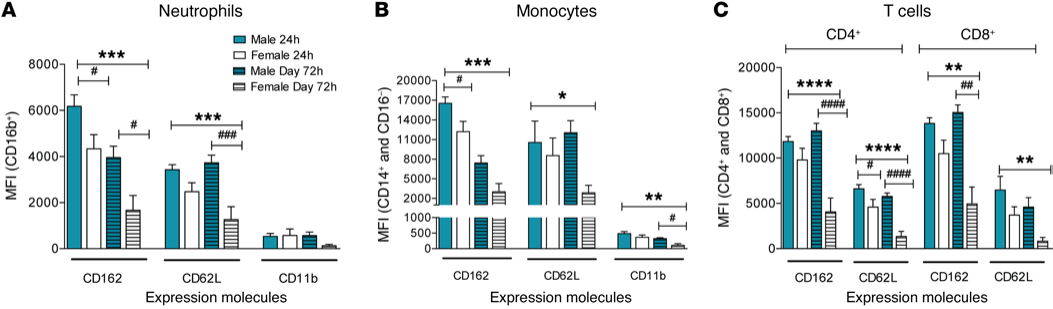
Reduced inflammatory cell activation state in cantharidin-induced blister exudates in female compared with male healthy volunteers. Mean fluorescence intensity (MFI) of the expression molecules CD162, CD62L, and CD11b on (**A**) neutrophils, (**B**) inflammatory monocytes, and (**C**) CD4^+^ and CD8^+^ T cells in healthy male (*n* = 16) and female (*n* = 16) volunteers. Data are shown as mean ± SEM. Statistical significances determined using 2-way ANOVA, *P < 0.05, **P < 0.01, ***P < 0.001, and ****P < 0.0001; followed by Šídák’s post tests, ^#^*P* < 0.05, ^##^*P* < 0.01, and ^####^*P* < 0.0001 comparing the sexes at each time point for all the panels.

Interestingly, as shown in Figure 5, irrespective of whether there were differences in the absolute numbers of specific cell subtypes between the sexes, all cell types showed a substantially and significantly reduced expression of all 3 activation markers in females compared with males. In 24-hour blister samples, using a cytokine/chemokine bead array, we identified no differences between the sexes in the levels of the proinflammatory cytokines/chemokines, which included IL-6, the neutrophil chemokines CCL5 and CXCL1, and the monocyte chemokine CCL2. Interestingly, we found higher levels of IL-8 in the blisters from female volunteers compared with males and importantly a trend for higher expression of the antiinflammatory cytokine IL-10 in females (Supplemental Table 4).

### Elevated levels of resolving mediators in female blister exudates.

**Figure 6:**
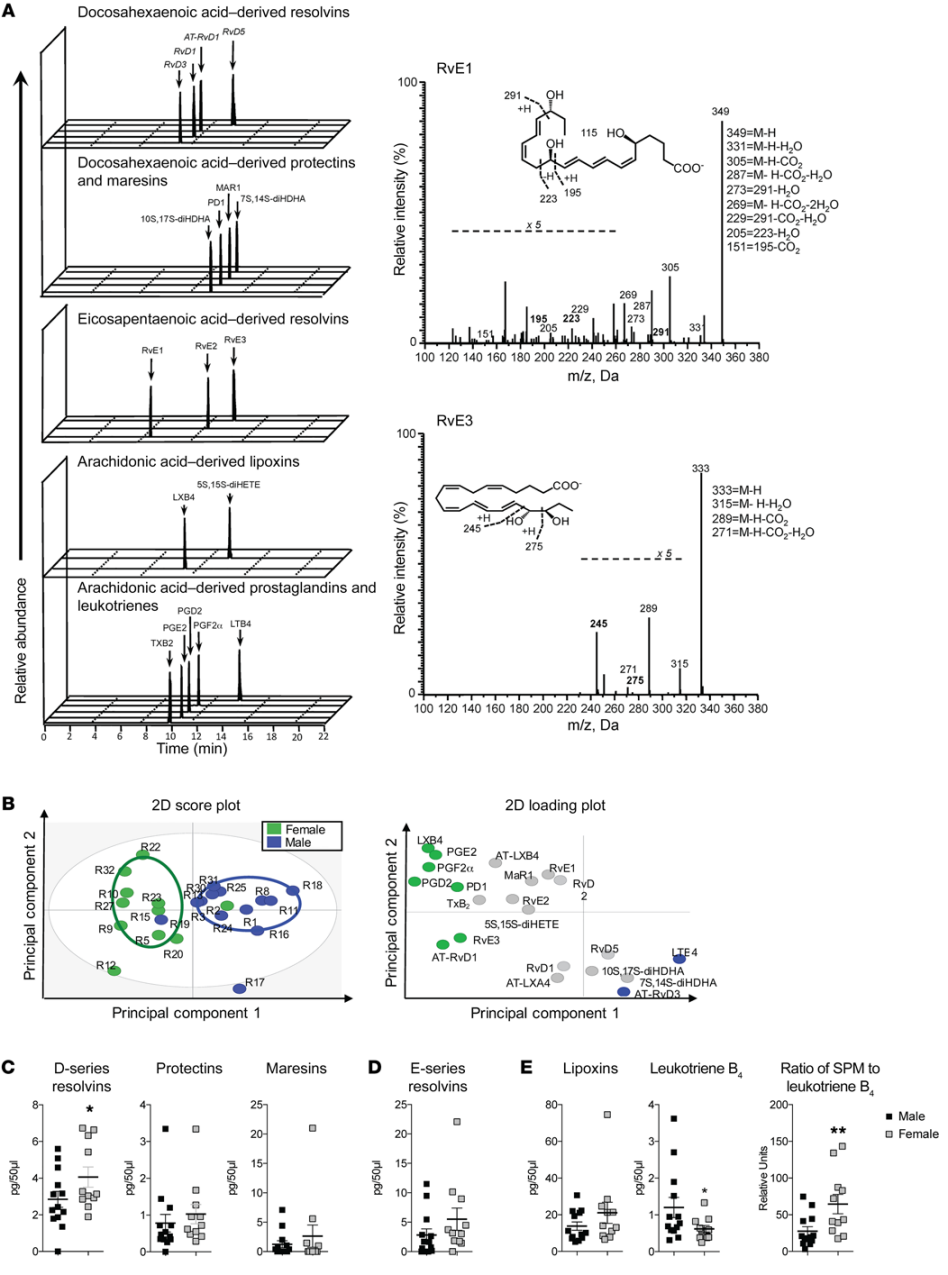
Female blister exudates display a pro-resolving mediator profile. Lipid mediators from exudates were extracted using C18 solid-phase extraction and profiled using LC-MS/MS–based lipid mediator profiling. (**A**) Representative multiple reaction monitoring chromatograms for identified lipid mediators and pathway markers from the 3 major bioactive metabolomes (left panel) and MS/MS spectra employed for their identification (right panel). (**B**) Partial least squares discriminant analysis of exudate lipid mediator profiles: left panel, 2D score plot; right panel, corresponding 2D loading plot. (**C**–**E**) Cumulative levels for the lipid mediator from the (**C**) docosahexaenoic acid, (**D**) eicosapentaenoic acid, and (**E**) arachidonic acid SPMs, arachidonic acid–derived LTB4, and ratio of SPM to LTB4. Results shown are mean ± SEM of *n* = 11 females and *n* = 13 males for **B**–**E**. Statistical significance determined using Students 2-tailed unpaired *t* test; *P < 0.05 for **C**–**E**.

Since lipid mediators play an important role in both the initiation and the termination of acute inflammation (26, 27), we next profiled lipid mediator levels in 24-hour blister exudates to investigate whether mediator levels from the three major bioactive metabolomes were differentially regulated between males and females. Using liquid chromatography–tandem mass spectrometry (LC-MS/MS) we identified mediators from the arachidonic acid (AA), eicosapentaenoic acid (EPA), and docosahexaenoic acid (DHA) bioactive metabolomes, including leukotriene B_4_ (LTB4), prostaglandin E2 (PGE2), resolvin D1 (RvD1), RvD2, RvD3, and protectin D1 (also known as PD1). These molecules were identified in accordance with published criteria (28), including matching retention times and >6 diagnostic ions in the MS/MS to authentic or synthetic standards (Figure 6A). Multiple reaction monitoring (MRM) was used to quantify mediators in these blister exudates. Using orthogonal partial least squares discriminant analysis (OPLS-DA), distinct clusters for lipid mediator profiles in the exudates were evident between male and females (Figure 6B and Supplemental Table 5). Assessment of overall levels of each of the lipid mediator families demonstrated that the specialized pro-resolving mediators (SPM) levels were elevated in female blister exudates when compared with those obtained from males (sum of all of the SPMs significantly greater in females, 17.2 ± 7.4 pg/50 μl [SD] than males, 10.5 ± 6.3 pg/50 μl; *P* = 0.015), with particularly the D-series resolvin levels collectively demonstrating a significant increase in females compared with males (Figure 6C). In these exudates we also found significantly higher levels of the potent leukocyte chemoattractant LTB4 in male exudates when compared with female exudates (Figure 6D), thus indicating that female-derived exudates gave an overall pro-resolving lipid mediator profile when compared with male exudates (Figure 6E). To determine whether similar differences were evident in volunteers subjected to typhoid vaccination, we assessed the levels of lipid mediators in plasma from volunteers 8 hours following vaccination, the time point at which the differences in endothelial function were most evident (Figure 3A). In plasma from these patients, we identified lipid mediators from all three major bioactive metabolomes, in accordance with published criteria (28). Multivariate analysis of lipid mediator profiles obtained using MRM gave two distinct clusters. Assessment of mediators that were associated with either of the clusters demonstrated that LTB4 was associated with the male cluster with a variable in importance score >1, whereas RvE1 and RvE3 were found to associate with the female clusters. Statistical analysis of each of the bioactive mediator families demonstrated that in females, there was a significant decrease in the LTB4 metabolome and a significant upregulation of the E-series resolvins specifically, RvE1 and RvE3 (see Supplemental Figure 6 and Supplemental Table 6).

## Discussion

It is accepted that premenopausal women have reduced levels of CVD compared with their age-matched counterparts (4). The exact mechanisms involved in mediating this protection are uncertain but have been attributed to an effect of female sex hormones (5). Separately, we know that while women tend to experience increased autoimmune disease, they have lower rates of inflammation in the context of chronic disease such as CVD and infection (29, 30). Since good evidence links innate immune responses with CVD (31) and inflammation is particularly thought to be causative in endothelial dysfunction (a critical pathogenic step in CVD progression), we speculated that reduced acute inflammatory responses in females may contribute to the protection of women against endothelial dysfunction and ultimately CVD development. Using experimental models to test this hypothesis in healthy volunteers, we show that female sex protects against the endothelial dysfunction induced by a mild systemic inflammatory response and that this protection likely relates to an enhanced leukocyte surveillance in the female circulation and an accelerated resolution of inflammation. We speculate that this enhanced capacity to deal with and then recover from an inflammatory stress likely plays a crucial role in the reduced rates of inflammatory CVD in premenopausal females.

Administration of typhoid vaccine to healthy volunteers causes a transient endothelial dysfunction reflected by a reduced response to reactive hyperemia (12). In this study, the FMD response was reduced following typhoid vaccination in males, but in contrast, in females FMD was greater following vaccine administration. Comparison of the change in FMD from baseline between the sexes (the primary outcome) demonstrates a clear protection in females from the damaging effects of systemic inflammation induced here by typhoid vaccination. Since responses to the endothelium-independent vasodilator GTN in both sexes were unchanged, nor were there any changes in arterial stiffness measures of PWV and PWA, these findings suggest that protection against the damaging effects of typhoid vaccination evident in females relates to alterations in endothelial function and not changes in smooth muscle reactivity. This latter observation agrees with previous findings demonstrating no effect of typhoid vaccination upon NO donor-induced brachial artery dilator response to either sublingual GTN (12) or intra-arterial infusions of sodium nitroprusside (32).

Plasma nitrite is thought to reflect endothelial NO generation, and NO is considered to mediate at least in part the shear stress-induced FMD response (33–36). Post hoc correlation analyses show that while FMD responses in females correlated directly with circulating nitrite levels, there was no such relationship in males. The primary pathway implicated in upregulation of beneficial NO levels in health is phosphorylation of eNOS via an AKT-dependent pathway; and this pathway underlies estrogen-induced enhanced NO biovailability (37, 38). It is likely that this pathway is responsible for the enhanced FMD responses in this study. Interestingly, in preclinical studies it has been suggested that in blood vessels of female animals, estrogen-induced upregulation of the AKT/eNOS phosphorylation pathway is resistant to repression by pathological stimuli that cause profound endothelial dysfunction in males (39). Additionally, evidence also demonstrates an increased efficacy of stimuli activating the AKT pathway in females compared with males (40). Of relevance to this study, acute low-grade inflammation, while exerting proinflammatory pathways, also triggers reflex protective effects particularly through upregulating AKT phosphorylation (41, 42). These observations together support the view that the enhanced FMD response in females following typhoid is likely secondary to a phosphorylation of eNOS resulting in greater shear stress–induced NO generation. The lack of a correlation between nitrite levels and FMD in males suggests that the FMD response in this sex is not entirely dependent upon NO (43). Indeed, the latter seems likely, since a recent meta-analysis of studies assessing the contribution of NO in mediating FMD estimated that 47%–67% of the response could be attributed to NO — the number varying according to the methodology used (43) but also possibly to the high inter-individual variability (44) — and now our data suggest that this also depends upon sex.

As expected, a number of demographic variables were distinct between the sexes despite being age-matched and healthy (45). These include BMI and blood pressure but also wbc count. The elevated wbc at baseline in females has been attributed to higher neutrophil numbers (46), and indeed in our study neutrophils accounted for the differences between men and women. Importantly, all women recruited into these studies attended experimental days at the midpoint of their menstrual cycles, when estrogen levels are generally at their highest and so also are the leukocyte and neutrophil counts (47) — the latter thought to be due to an action of estrogen. This raised wbc/neutrophil number in females may be due to estrogen-induced margination of neutrophils from the bone marrow. In healthy women asked to conduct controlled exercise, as a trigger for changes in granulocyte distribution, the response remained unchanged at different stages of the menstrual cycle (47). However, studies in mice suggest that low-dose estrogen inhibits rather than triggers bone marrow margination (48). Exactly why these diametrically opposing results occur is uncertain, but they may simply reflect important species differences. Interestingly, while we saw no significant differences in monocyte numbers at baseline, our data suggest a generalized raised activation state of circulating neutrophils and monocytes in females compared with males. In particular, we saw enhanced expression of both CD162 and CD62L adhesion molecules on these cell types in men at 8 hours, with recovery of baseline expression levels by 32 hours following typhoid vaccine, with no significant changes in expression levels in women. If anything there was some suggestion of a slight but nonsignificant decrease in females in the expression of the adhesion molecules relative to baseline. With respect to CD62L, it is possible that this reflects the shedding of CD62L that occurs during inflammatory responses and is thought to enhance leukocyte transmigration (49); however, further experiments powered against CD62L levels are needed investigate this. In addition, both cellular CD162 and CD62L have been identified as critical mediators of the homing, rolling, and adhesion of both neutrophils and monocytes to sites of inflammation through interaction with the endothelium, and thus the enhanced cell expression in the males is likely to reflect an increased cell activation (21, 22).

Irrespective of which mechanisms might underlie the differences, the outcome of such a response would be to reduce exposure of the vasculature to the potentially long-term detrimental effects of a sustained inflammatory response, and this offers a possible explanation for the absence of vascular dysfunction in the female sex. Interestingly, recent evidence suggests the sex differences in leukocyte count and activation state evident in healthy volunteers are lost in individuals with raised CVD risk, although it is worth noting that the average age of the women in this particular study was 62, an age when the majority of the women are likely to have been postmenopausal (although whether this was the case is unclear) (50). Our observations in this systemic inflammation model suggest that there is an enhanced surveillance and readiness to deal with inflammatory stimuli resulting ultimately in a reduced detrimental impact upon the vasculature. In order to probe the possible mechanisms further, we assessed inflammatory responses using the cantharidin-based model of innate inflammatory response (20).

Cantharidin is a protein phosphatase 1 and 2 alpha inhibitor (51). When applied to the skin, it results in acantholysis and blister formation, which has been attributed to the detachment of tonofilaments from desmosomes (52). This in turn causes leukocyte extravasation, cytokine release, and clinical inflammation. During inflammation, leukocytes and proteins traverse the blood vessel into the extravascular space. Recruitment of cells to inflammatory sites is dependent on the release of vasoactive and chemotactic factors that increase regional blood flow, increase microvascular permeability, and promote the exudation of leukocytes from the circulation into the tissues (53). Cantharidin was used in this study to trigger this response to enable quantification of and therefore comparison between the sexes of this inflammatory response through assessment of the amount of fluid collecting into the blister, as well as characterizing the number and types of inflammatory cell collecting within the exudate. In this study, cantharidin elicited blister responses in both males and females of a magnitude similar to that reported previously, in terms of edema (i.e., volume) and total number of cells (25). Indeed, at the 24-hour time point, the number of neutrophils, the first cell recruited to a site of inflammation, was very similar between the sexes, suggesting that female sex does not suppress the capacity to respond to an inflammatory stimulus per se. Assessment of the proinflammatory cytokine/chemokine profile in the main supports this view. Our analyses suggest no differences between the sexes of some of the key mediators previously implicated in the cantharidin blister response in humans, including IL-6 (25), neutrophil (CXCL1, CCL5), and monocyte chemokines (CCL2) (54). However, at 72 hours there was a pronounced reduction in the blister volume, with a slight trend toward reduced cell numbers in females — a reduction accounted for by a pronounced and significant reduction in inflammatory monocyte numbers. Importantly, in many of the women (~70% vs. 30% in men), the blisters had resolved by 72 hours, resulting in no volume collection at all. Since the immediate (24-hour) response was similar between the sexes, we speculated that the reduced cell number and volume at 72 hours likely reflects an enhanced rate of resolution of inflammation. This is in line with recent evidence demonstrating the key role of resolvins in clearing edema following an inflammatory insult, where edema in the lung induced in mice with hydrochloric acid was reduced by treatment with aspirin-trigged RvD3 (55). To explore this possibility, we assessed the level of IL-10, a pivotal cytokine released during the resolution stage of an inflammatory response that is derived from the antiinflammatory intermediate (M2) monocyte (56). We did observe a trend toward enhanced IL-10 in the blisters of females at 24 hours; however, this did not reach statistical significance, likely due to the fact that the study was not powered for this measurement. However, it is thought that IL-10 represses further cell recruitment in part by downregulating inflammatory cell activation. Indeed, the levels of CD62L, CD11b, and CD162 expression were all substantially reduced, supporting the view that while the response to the inflammatory stimulus was similar between the sexes, in females this inflammatory response was likely cleared and “resolved” at a much faster rate.

It is now accepted that the resolution of acute inflammation is an active process that is initiated when the very first leukocytes arrive at the inflammatory site. This process of resolution is triggered by the recruited cells themselves and is mediated by a switch in the local production of lipid mediators from the proinflammatory eicosanoids, including prostaglandins and leukotrienes, to the pro-resolving and tissue-reparative SPMs, including the lipoxins and resolvins (26). Using targeted lipid mediator profiling, we found a 1.4- to 2-fold-higher level for each class of SPMs in females. In addition, SPM amounts relative to those of the proinflammatory/chemotactic lipid mediator LTB4 exposed a SPMs/LTB4 ratio in the females that was 3 times greater than in males, suggesting that the balance of pro-resolving to proinflammatory lipid mediators was tipped in favor of resolution in females. SPMs share a number of key defining bioactions, including their ability to limit neutrophil recruitment to the site, counterregulate the production and actions of proinflammatory mediators, and promote macrophage phagocytosis of cellular debris and apoptotic cells. In addition, each SPM displays unique biological actions; for example, MAR1 is produced in the later stages of the resolution phase and promotes tissue repair as well as displaying potent antinociceptive actions. RvD1, RvD5, and PD1 are produced during self-limited infections and promote the clearance of bacterial infections, while resolvin D2 potently regulates endothelial NO production. In the present study, we found a significant reduction in both neutrophil and monocyte/macrophage activation state in females compared with males. This is in line with recent findings that demonstrate that RvD1 potently regulates neutrophil recruitment and adhesion molecule expression (54), as well as with the finding that RvD1 regulates LTB4 formation by regulating 5-lipoxygenase phosphorylation and translocation to the nuclear membrane (57). This balance in favor of the resolving SPMs in the blisters of females compared with males was also evident in the plasma of females 8 hours following typhoid vaccine, supporting the view that active resolution is likely to underlie the protection against vascular dysfunction in females. Of note, in these plasma samples, we found an upregulation of molecules from the E-series resolvins that carry potent cardiovascular protective actions (58) The differential regulation of EPA- versus DHA-derived resolvins in plasma and inflammatory exudates may reflect the activation of different biosynthetic pathways by the different stimuli, in line with published findings (59), as well as a differential utilization of precursor fatty acids in plasma versus tissues (28, 60).

Interestingly, this pathway has also been proposed to be a target for sex steroid activity. Studies in vitro using whole blood and isolated neutrophils collected from healthy male and female volunteers demonstrated that following stimulation with a proinflammatory stimulus, the levels of the 5-lipoxygenase products, including LTB4, were reduced in males compared with females and that this effect related to activity of testosterone and the inhibition of nuclear localization of 5-lipoxygenase (61). Indeed, treatment of neutrophils collected from healthy women with 5-α-dihydrotesterone lowered the levels of 5-lipoxygenase products produced following stimulation to those evident in the cells isolated from males (61). Of note, treatment of neutrophils from donors of either sex with female sex hormones, 17β-estradiol or progesterone, did not alter the levels of the proinflammatory lipid mediators. However, it is worth noting that for cells collected from female donors, the addition of female sex hormones in vitro may not have been effective since the cells will have been exposed to physiological levels of these hormone in vivo. Furthermore, because the levels of 5-lipoxygenase were low at baseline in cells of male donors, it is unlikely that with in vitro treatment they would be further inhibited. Treatment with additional 5-α-dihydrotesterone had no further effect in cells isolated from males, suggesting likelihood of this hypothesis. Exactly why in our studies we saw higher SPM/LTB4 ratios in females while in the study mentioned above higher ratios were evident in males is uncertain, but this may reflect the difference between in vivo and in vitro assessment, and further investigation is warranted. Additionally, further studies exploring the regulation of SPM receptors on inflammatory cells themselves and the impact of inhibiting SPM formation on resolution of inflammatory responses, as well as studies assessing the impact of raising SPM levels, are important to confirm the sexual dimorphism described but also to ascertain whether delivery of SPMs, particularly through dietary provision of substrate (27, 62), might offer a therapeutic approach that could be useful in limiting inflammatory responses.

### Limitations.

There are a number of limitations of our work. First, both models of inflammation provide an approximation of the systemic and local inflammatory scenarios. It is possible that the specific cellular profile induced by typhoid or cantharidin are not the same as those induced in a CVD scenario.

Since a large number of female blisters had resolved at the 72-hour time point, a full profile of the resolution time course was not possible. Future experiments including a time point midway between 24 and 72 hours might provide a more detailed window on the time course of the resolution between the sexes. In addition, recording of the time of resolution in males would also provide an improved comparison of the time scale between the sexes.

### Conclusions.

We have demonstrated that the damaging effects of systemic inflammatory stimuli on the vasculature are limited in females and that this may be due to a more rapid resolution of the local inflammatory response. Strategies directly targeting resolution may be useful in limiting the damaging effects associated with the raised systemic inflammation that is characteristic of early pathogenesis in CVD.

## Methods

### Volunteers.

Equal numbers of male and female volunteers were recruited. Volunteers were included if they fulfilled the following inclusion criteria: 18–45 years of age, BMI of 18–40 kg/m^2^, and no typhoid vaccination in the previous 2 years. Exclusion criteria included: subjects with a history of hypertension, diabetes, or hypertensive on BP measurement; subjects who were pregnant or any possibility that a subject may be pregnant, unless in the latter case a pregnancy test is performed with a negative result; subjects with a history of any serious illnesses, including recent infections or trauma; subjects taking systemic medication (other than the oral contraceptive pill); subjects with self-reported use of mouthwash or tongue scrapes; subjects with recent or current antibiotic use; subjects with a history or recent treatment (within the preceding 3 months) of any oral condition (excluding caries), including gingivitis, periodontitis, and halitosis; and subjects who have recently participated (preceding 3 months) in any clinical studies involving administration of an inflammogen. Female volunteers appointments were scheduled to fall at a maximum of 2 weeks and no less than 1 week prior to the due date of their next menstruation when estrogen levels at their highest.

All clinical studies were performed in a quiet temperature-controlled laboratory (24°C–26°C), and individuals were studied at the same time of day during the 3 days they were involved in the study period. Volunteers refrained from caffeine consumption and strenuous exercise the day before and the day of the respective study, and were fasted overnight before all study visits.

### Study 1: typhoid-induced systemic inflammation study.

A total of 24 healthy volunteers (12 males and 12 females) were consented for this 3-day study. On day 1 volunteers attended the clinic at 3 p.m. and measured their own blood pressure using a portable blood pressure device. Body temperature was measured using a standard Braun Thermoscan Ear Thermometer. Following this, PWA, PWV, and FMD were determined. Blood was then collected. On day 2, the volunteers attended to receive the typhoid vaccination into the gluteal or deltoid region at 8 a.m. and returned at 3 p.m. for a repeat of all the previous measurements. On day 3, volunteers attended the clinic at 3 p.m. for the final time and had a final repeat of all measurements (Figure 1A). In a separate study, we recruited a further 16 healthy volunteers — 8 males and 8 females — to assess endothelium-independent vasodilation using glyceryl trinitrate (GTN) as per previous recommendations (63). Briefly, the volunteers attended the clinic as above but received a sublingual (0.4 mg) dose of GTN spray (Nitrolingual Pump Spray, Merck) in place of the reactive hyperemia stimulus. Whole blood differentials and flow cytometry were measured to assess both leukocyte and platelet numbers and activation state, and plasma was collected for assessment of NOx levels and CRP.

### Study 2: cantharidin-induced blister study.

A total of 32 healthy volunteers (16 male and 16 female) were consented for this 4-day study. On day 1, the volunteers measured their own blood pressure using a portable blood pressure device. After this, cantharidin solution was applied to the ventral aspect of a forearm. Blood was then collected. On day 3 (48 hours after the initial cantharidin application), volunteers returned to have cantharidin applied to a separate area, no less than 5 cm away from the initial site. Finally, on day 4 (72 hours after the initial cantharidin application), the volunteers measured their blood pressure again, and then blister fluid was sampled from both blisters, and blood, urine, and saliva samples were collected. Blister fluid volume, cell count, flow cytometric analysis of cell type and activation state, and cytokine/chemokine analysis were also run.

For the application of cantharidin solution, 10 μl cantharidin (cantharone 0.1%, Dormer Laboratories) was applied to filter paper discs and placed on the ventral aspects of the forearm as previously described (48–51) and dressed appropriately. Blister fluid was then harvested at 24 hours (acute phase) and 72 hours (resolution phase) by carefully piercing the side of the blister with a 25-gauge needle and rolling a pipette tip over the surface of the blister to express blister fluid, which was then collected in a siliconized pipette; the fluid was then stored on ice until further analysis.

### Blood sampling.

Blood was collected into EDTA tubes for differential count analysis conducted by St. Bartholomew’s Hospital Haematology Department. A further 4 ml of blood was collected in 1.8 mg EDTA per ml of blood for NOx measurement, and another 4 ml in 3.2% buffered sodium citrate for aggregation assays and platelet flow cytometry. All blood was collected into Vacutainers (BD Biosciences) through a 21-gauge butterfly needle inserted into an antecubital vein. For measurement of NOx levels, blood samples were centrifuged immediately (1,300 *g*, 4°C, 10 minutes), and the supernatant was collected and stored at –80 °C pending analysis by ozone chemiluminescence as previously described (64, 65).

### Blood pressure measurement.

Blood pressure was measured at baseline to confirm healthy volunteer status. An Omron 705IT was used for all BP measurements while participants were seated, and readings were performed in triplicate according to established guidelines (66). Laminated coverings were used for the machine and the printer so that both investigators and participants were blinded to the readings. The means of the second and third readings were used to calculate the final BP measurement.

### PWV and PWA measurements.

A Vicorder device (Skidmore Medical Ltd.) was used to simultaneously record the pulse wave from the carotid and femoral sites using an oscillometric method. A small, inflatable neck pad is placed directly over a single carotid artery and secured around the neck by a Velcro tab. A cuff is placed around the subject’s ipsilateral upper thigh. Both carotid and femoral cuffs are inflated automatically to 65 mmHg, and the corresponding oscillometric signal from each cuff is digitally analyzed to extract the pulse time delay. The distance between the sternal notch and the thigh cuff is measured and used as a standard estimate for the aortic length. From these measurements aortic PWV can be derived as PWV = aortic distance/pulse time delay (67). PWA was measured as follows: After 5-minute supine rest, a 100-mm inflatable cuff was attached to the non-dominant arm and statically inflated to 65 mmHg. Brachial artery waveforms were digitally computed by Vicorder using a volume-displacement method for at least 10 cardiac cycles to calculate augmentation index.

### Measurement of brachial artery diameter.

Brachial artery diameter in the non-dominant arm was measured with high-resolution external vascular ultrasound (Acuson 128XP/10 with a 7.0-MHz linear array transducer). The vessel was scanned in longitudinal section, and the center was identified when the clearest views of the anterior and posterior artery walls had been obtained. Images were magnified with a resolution box function and gated with the R wave of the ECG. End-diastolic images of the artery were acquired every 3 seconds with customized data acquisition software and stored in digital format offline for later analysis (34). Brachial artery diameter was measured continuously for 1 minute at baseline, during 5 minutes of reduced blood flow (induced by inflation to 300 mmHg of a pneumatic cuff placed at a site distal to the segment of artery being analyzed), and for a further 5 minutes during reactive hyperemia after cuff release. Brachial artery diameter and brachial artery dilation expressed as percentage and absolute increase from baseline diameter and time to peak dilatation were determined using the software Vascular Analysis Tools (Medical Imaging Applications LLC).

### Measurement of antibody titer.

Plasma samples taken at baseline and at 32 hours after typhoid were analyzed for antibody titer using a commercially available kit (Typhoid Vi IgM ELISA kit; Alpha Diagnostic International) as per the manufacturer’s instructions.

### Platelet flow cytometry.

Whole blood flow cytometry was used to measure platelet P-selectin with the use of a modification of previously published protocols and recommendations (68). The platelet population was identified via labeling with a CD42b (1:25; catalog 11-0429-71, eBioscience) monoclonal antibody conjugated to allophycocyanin and a CD62P (P-selectin; catalog MCA883F, AbD Serotec) monoclonal antibody conjugated to fluorescein isothiocyanate, which was used to determine the P-selectin expression. Isotype controls for CD62P and CD42b were used to ensure antibody specificity. Samples were incubated at room temperature for 20 minutes with PBS, ADP (3 μmol/l or 10 μmol/l; Labmedics), and collagen (1 μg/ml or 3 μg/ml; Takeda) before fixing with 1% paraformaldehyde (Sigma-Aldrich) and analyzed with the use of a Becton Dickinson LSR Fortessa Cell Analyzer. A total of 10,000 platelets were acquired in the CD42b-positive region. Results were expressed as the percentage of platelets positive for P-selectin (69).

### Platelet-monocyte aggregate expression.

Platelet-monocyte aggregates were determined with the use of a modification of previously published protocols with antibodies that were selective for platelet CD42b, and monocyte marker CD14 (catalog 55397, BD Biosciences) samples were analyzed with the use of a Becton Dickinson LSR Fortessa Cell Analyzer. Isotype controls were conducted.

### Platelet aggregation.

Platelet aggregation was assessed in whole blood in response to collagen (1–3 μg/ml), ADP (3 or 10 μM), and PBS (as control) using an impedance aggregometer (Multiplate analyzer, Dynabyte Medical) measured over a 6-minute period. Aggregation was quantified as AUC, giving a measure of total resistance Ω * time. Briefly, 300 μl of citrated whole blood was added to 300 μl of normal saline with 3 mM CaCl_2_ (Sigma-Aldrich) and equilibrated with constant magnetic stirring for 3 minutes prior to addition of agonist and platelet aggregation measurement.

### Inflammatory cell cytometry.

The systemic inflammatory response at baseline and after typhoid vaccination was assessed using flow cytometry. Whole citrated blood (50 μl) was incubated with specific monoclonal antibodies conjugated to fluorochromes to identify lymphocytes, monocytes, and neutrophils (CD14, CD16 catalog 555407, CD16b catalog 550868, BD Biosciences; CD3 catalog 11-0039-42, CD4 catalog 25-0049-42, CD8 catalog 45-00988-42, eBioscience), as well as for determination of cell activation state via measurement of the expression of CD162 (catalog 17-1629-42), CD62L (catalog 47-0629-42, eBioscience), and CD11b (catalog 558123, BD Bioscience) (and their isotype controls) on the cell surface. Samples were mixed gently and then allowed to incubate for 30 minutes before removal of excess antibody with PBS and centrifugation for 5 minutes, 400 *g* at 4°C. After decanting the supernatant, whole blood lysing reagent (Beckman Coulter) was added to each tube. After giving each tube a thorough vortex, a formaldehyde-containing fixative agent was added. The samples were washed twice using PBS and centrifuged for 5 minutes, 400 *g* at 21°C. Finally, 1% paraformaldehyde (Sigma-Aldrich) was added to the tubes. All samples were then analyzed using a Becton Dickinson LSR Fortessa Cell Analyzer and recorded with BD FACSDiva software.

For blister fluid prior to flow cytometry a total cell count was determined using a hemocytometer. The fluid was then centrifuged for 5 minutes, 400 *g* at 4°C. After decanting the supernatant, the leukocyte pellet was resuspended in FACS buffer to ensure between 50,000 and 100,000 cells/20 μl. 20 μl of the resuspended cells were then incubated with monoclonal antibodies and analyzed as above.

### Lipid mediator profiling.

Standards for LC-MS/MS profiling were purchased from Cayman Chemical or provided by Charles N. Serhan (Harvard Medical School, Boston, Massachusetts, USA; supported by NIH-funded P01GM095467). Blister exudates or plasma samples were added to 2 volumes of ice-cold MeOH containing deuterated internal standards (d_4_-LTB4, d_8_-5S-HETE, d_4_-PGE2, d_5_-LXA4, and d_5_-RvD2, 500 pg each). These were then kept at –20°C for 45 minutes to allow for protein precipitation and subjected to solid-phase extraction as per ref. 28. Methyl formate fractions were then brought to dryness using a TurboVap LV (Biotage) and products suspended in water-methanol (50:50 vol/vol) for LC-MS/MS. A Shimadzu LC-20AD HPLC and a Shimadzu SIL-20AC autoinjector, paired with a QTrap 5500 (ABSciex) were utilized and operated as described previously (28). To monitor each lipid mediator and respective pathways, an MRM method was developed with diagnostic ion fragments and identification using recently published criteria (28), including matching retention time to synthetic and authentic materials and at least 6 diagnostic ions for each lipid mediator. Calibration curves were obtained for each using authentic compound mixtures and deuterium-labeled lipid mediator at 0.78, 1.56, 3.12, 6.25, 12.5, 25, 50, 100, and 200 pg. Linear calibration curves were obtained for each lipid mediator, which gave *r^2^* values of 0.98–0.99. All measurements were conducted in a blinded manner.

### Cytokine/chemokine analysis.

Cytokine/chemokine expression profiles were analyzed using a customized bead array (IDEXX ProCyte Dx Hematology Analyzer) to measure IL-6, IL-8, IL-10, CXCL1, CXCL2, CCL5, and CCL2. All measurements were conducted in a blinded manner.

### Power and statistical analysis.

Based upon previous observations demonstrating typhoid vaccine–induced endothelial dysfunction (12). The primary outcome measure was change in % FMD, with an expected difference between the sexes of 1.5% and an SD of 1.5. Twelve subjects in each group were required to achieve 80% power.

For the cantharidin study, based upon previous observations demonstrating that cantharidin triggers a total cell response of 0.6 × 10^6^ cells/ml with an SD of 0.2 (70) and in preclinical models where leukocyte recruitment was reduced by 50% in females (8), with a power of 0.9 a total of 12 individuals are required for each sex for statistical power. However, due to the limited sample size available for all analyses, we prespecified an *n* = 16 for each group to enable sufficient independent samples to assess soluble mediators. Where *n* values shown are less than 16, this is due to insufficient sample size for full analysis.

Data are shown as mean ± SD. Comparisons between the sexes have been made using unpaired 2-tailed *t* tests or 2-way ANOVA as appropriate. With respect to the latter, post hoc comparisons were made if the F statistic achieved *P* < 0.05 and there was no significant variance in homogeneity between groups using Holm-Šídák’s multiple comparison tests. For multivariate statistical analysis, OPLS-DA (71) was performed using SIMCA 13.0.3 software (Umetrics) following unit variance scaling of linear model (LM) amounts. OPLS-DA is based on a linear multivariate model that identifies variables that contribute to class separation of observations (blister exudates) on the basis of their variables (LM levels). During classification, observations were projected onto their respective class model (males vs. females). The score plot illustrates the systematic clusters among the observations (closer plots presenting greater similarity in the data matrix). Loading plot interpretation identified the variables with the best discriminatory power (variable importance in projection close to or greater than 1) that were associated with males (blue) or females (red) and contributed to the tight clusters observed in the score plot.

### Trial approval and registration.

The studies were peer reviewed by the institutional review board and were granted full ethics approval by National Research Ethics Service (NRES) Committee London — City Road and Hampstead (11/LO/2038). Informed, written consent was taken after participants satisfied the inclusion criteria. The studies have been registered on ClinicalTrials.gov (NCT01582321).

## Author contributions

KSR conducted studies in healthy volunteers, analyzed data, and contributed to writing of the manuscript. VK conducted studies in healthy volunteers, analyzed data, designed experiments, and contributed to writing of the manuscript. SV conducted studies in healthy volunteers and analyzed data. RSK acquired data. US conducted studies. SK conducted studies. SVE acquired and analyzed data. LCG acquired and analyzed data. JB conducted studies. KP conducted studies. CS conducted studies. FD provided technical support. RAC acquired data. FMB analyzed data and provided advice. JD acquired and analyzed data and contributed to writing of the manuscript. AA designed the research studies, analyzed data, and wrote the manuscript.

## Supplementary Material

Supplemental data

ICMJE disclosure forms

## Figures and Tables

**Table 2:**
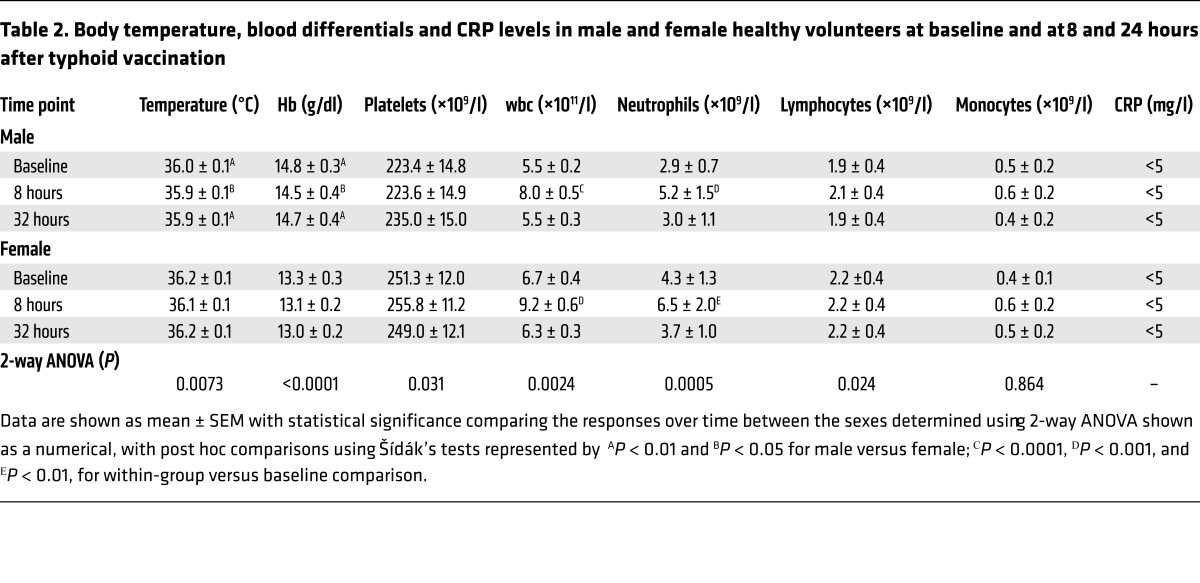
Body temperature, blood differentials and CRP levels in male and female healthy volunteers at baseline and at 8 and 24 hours after typhoid vaccination

**Table 1:**
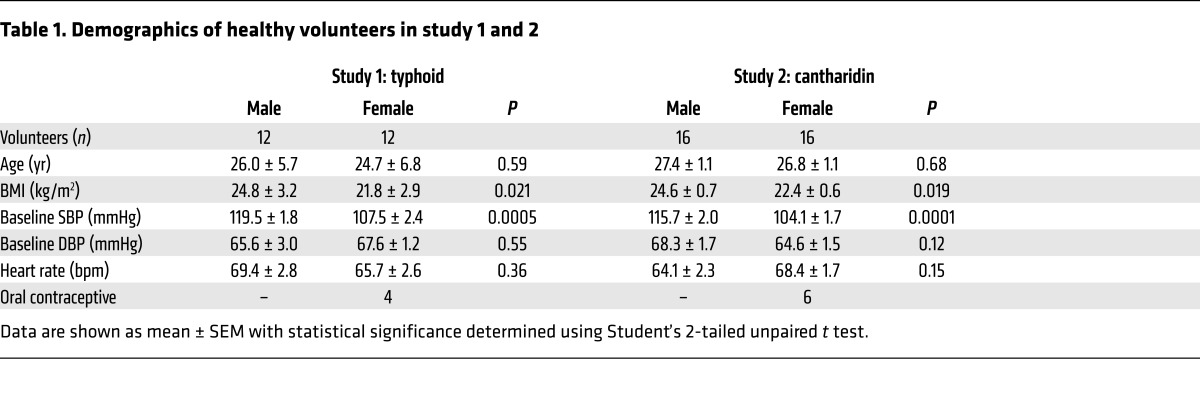
Demographics of healthy volunteers in study 1 and 2
