# Accelerated resolution of inflammation underlies sex differences in inflammatory responses in humans

**DOI:** 10.1172/JCI168068

**Published:** 2023-01-17

**Authors:** Krishnaraj S. Rathod, Vikas Kapil, Shanti Velmurugan, Rayomand S. Khambata, Umme Siddique, Saima Khan, Sven Van Eijl, Lorna C. Gee, Jascharanpreet Bansal, Kavi Pitrola, Christopher Shaw, Fulvio D’Acquisto, Romain A. Colas, Federica Marelli-Berg, Jesmond Dalli, Amrita Ahluwalia

Original citation: *J Clin Invest*. 2017;127(1):169–182. https://doi.org/10.1172/JCI89429

Citation for this corrigendum: *J Clin Invest*. 2023;133(2):e168068. https://doi.org/10.1172/JCI168068

The authors recently became aware that representative illustrations presented in Figure 6A and Supplemental Figure 6A might be mistaken for original data. As these schematics were used strictly for demonstrative purposes, they have been removed from the figure for clarity. Tabular representations of the experimental findings are provided in Supplemental Table 5 and Supplemental Table 6.

The updated Figure 6A and the legend for Figure 6 appear below. The supplemental document has been updated online.

## Figures and Tables

**Figure 6:**
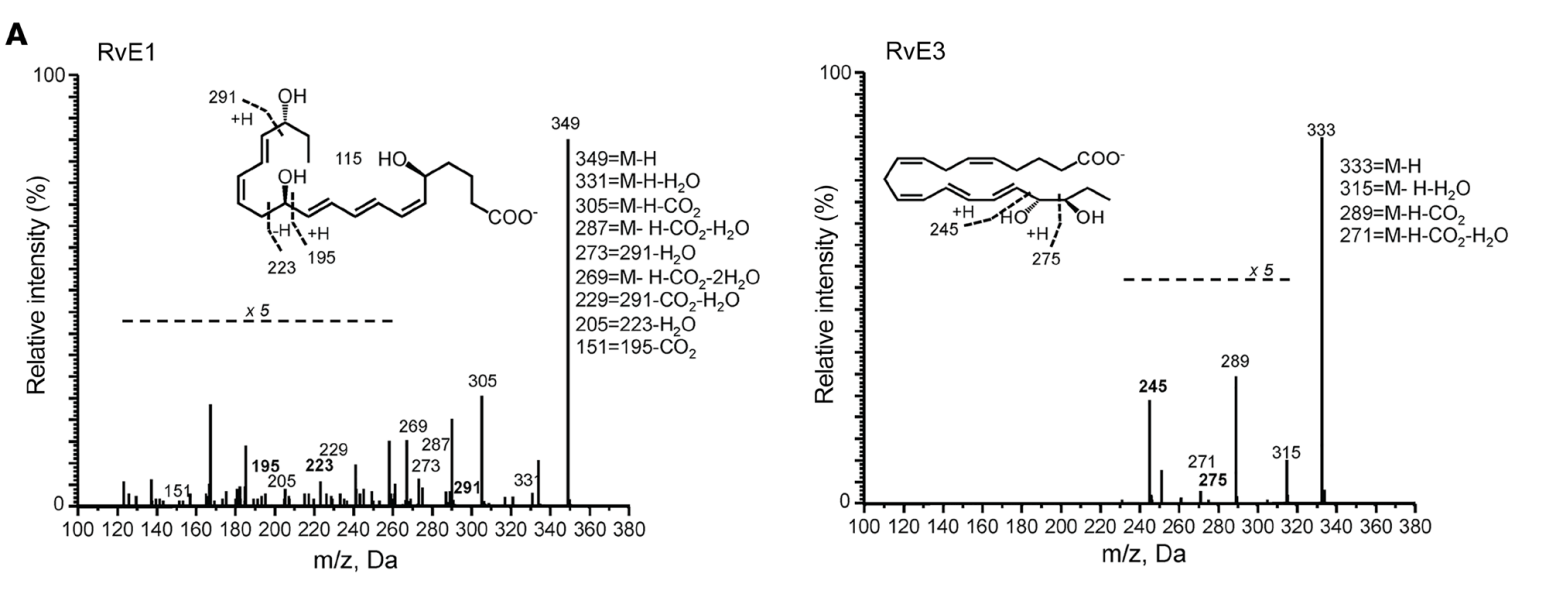
Female blister exudates display a pro-resolving mediator profile. Lipid mediators from exudates were extracted using C18 solid-phase extraction and profiled using LC-MS/MS–based lipid mediator profiling. (**A**) MS/MS spectra employed for identification of lipid mediators. (**B**) Partial least squares discriminant analysis of exudate lipid mediator profiles: left panel, 2D score plot; right panel, corresponding 2D loading plot. (**C**–**E**) Cumulative levels for the lipid mediator from the (**C**) docosahexaenoic acid, (**D**) eicosapentaenoic acid, and (**E**) arachidonic acid SPMs, arachidonic acid–derived LTB4, and ratio of SPM to LTB4. Results shown are mean ± SEM of *n* = 11 females and *n* = 13 males for **B**–**E**. Statistical significance determined using Student’s 2-tailed unpaired *t* test; *P < 0.05 for **C**–**E**.

## Version 1 01/17/2023

Electronic publication

