# Supplementary material for: Accelerated resolution of inflammation underlies sex differences in inflammatory responses in humans
Source: J Clin Invest. 2016 Nov 28;127(1):169–82. doi: 10.1172/JCI89429 (PMC5199722; doi:10.1172/JCI89429)

Supplementary Information

Accelerated resolution of inflammation underlies sex differences in inflammatory responses in humans

Krishnaraj S. Rathod, Vikas Kapil, Shanti Velmurugan, Rayomand S. Khambata, Umme Siddique, Saima Khan, Sven Van Eijl,, Jascharanpreet Bansal, Kavi Pitrola, Christopher Shaw, Romain A. Collas, Federica Marelli-Berg, Jesmond Dalli, Amrita Ahluwalia *

*** Corresponding author:**

Professor Amrita Ahluwalia
Prof of Vascular Pharmacology
Barts & The London Medical School
Queen Mary University of London
Charterhouse Square,
London, EC1M 6BQ
Email: a.ahluwalia@qmul.ac.uk
Tel: +44 207 882 8377
Fax: +44 207 882 5619

Supplementary Tables

Table S1

The effect of typhoid on the expression of platelet activation markers and platelet activity in healthy volunteers. Data are shown for % P-selectin expression in males and females following PBS treatment and in response to ADP (3 and 10 µM) or collagen (3 and 10 µg/ml) at baseline, 8 hours and 32 hours post vaccine. For % platelet monocyte aggregates levels were measured at baseline, 8 hours and 32 hours post-vaccine. Data are shown as mean ± s.e.mean and statistical analysis conducted using two-way ANOVA to determine the influence of sex for n=10 male and n=12 female volunteers (2 samples lost due to technical difficulties). Statistical significance was tested using two-way ANOVA with Sidak's post-hoc analysis.

	Males			Females			Sex comparison
	**Baseline**	**8 hours**	**32 hours**	**Baseline**	**8 hours**	**32 hours**	**P Value**
**% P-Selectin expression**							
**PBS**	2.3 ± 1.4	0.7 ± 0.2	2.2 ± 1.2	1.0 ± 0.2	0.5 ± 0.1	0.8 ± 0.3	0.517
**Collagen 1 µg/ml**	2.2 ± 0.7	1.3 ± 0.3	2.6 ± 1.1	2.3 ± 0.7	1.1 ± 0.2	1.2 ± 0.2	0.567
**Collagen 3 µg/ml**	3.7 ± 0.9	3.2 ± 0.8	4.2 ± 1.6	3.8 ± 1.3	2.7 ± 0.6	2.2 ±0.4	0.448
**ADP 3 µM**	9.1 ± 2.6	6.8 ± 1.3	4.2 ± 1.6	8.9 ± 2.1	12.3 ± 3.7	10.8 ± 3.3	0.723
**ADP 10 µM**	10.9 ± 3.3	7.1 ± 1.8	12.5 ± 3.3	12.0 ± 2.5	12.9 ± 3.0	13.8 ± 3.6	0.587
**% PMA expression**	31.5 ± 4.2	34.2 ± 6.4	26.8 ± 4.3	27.8 ± 5.4	34.4 ± 6.0	33.1 ± 4.7	0.611
**Platelet Aggregation (AU)**							
**PBS**	7.2 ± 2.1	8.8 ± 3.0	8.2 ± 2.3	9.2 ± 2.0	13.5 ± 2.5	8.1 ± 2.3	0.268
**Collagen 1 µg/ml**	59.5 ± 3.4	61.6 ± 5.2	54.8 ± 4.0	60.2 ± 5.3	74.6 ± 5.1	61.3 ± 5.2	0.086
**Collagen 3 µg/ml**	78.9 ± 3.7	91.5 ± 4.9	75.1 ± 3.9	83.8 ± 5.9	99.6 ± 5.9	76.8 ± 6.5	0.245
**ADP 3 µM**	35.1 ± 4.1	37.8 ± 4.1	37.7 ± 5.4	46.3 ± 5.8	54.4 ± 5.1	43.2 ± 5.5	0.008
**ADP 10 µM**	47.8 ± 4.1	49.9 ± 4.3	43.9 ± 3.7	57.0 ± 4.4	68.6 ± 3.5	56.9 ± 4.8	0.0002

Table S2

Vascular measures at baseline and at 8 and 32 hours following typhoid vaccination Data are shown as mean ± SD with statistical significance determined using using two-way ANOVA with post-hoc Sidak's tests shown as * for P<0.05, **P <0.01, ***P <0.001, ****P <0.0001 for comparison of time-points between the sexes and no significant differences for within group comparisons to baseline.

	Males (n=12)			Females (n=12)			
	**Baseline**	**8 hours**	**32 hours**	**Baseline**	**8 hours**	**32 hours**	**Sex comparison**
**Ultrasound**							
**Baseline brachial artery diameter, mm**	3.8 ± 0.5***	3.8 ± 0.5****	3.8 ± 0.4**	3.1 ± 0.3	3.1 ± 0.4	3.2 ± 0.4	<0.0001
**Time to peak diameter, min**	6.7 ± 0.5	6.7 ± 0.7	6.7 ± 0.8	6.7 ± 0.2	6.8 ± 0.2	6.8 ± 0.2	0.492
**PWA**							
**Augmentation index, %**	13.3 ± 1.9	13.8 ± 1.2	13.5 ± 1.7*	12.1 ± 1.4	11.6 ± 2.2	11.9 ± 1.9	0.24
**PWV, m/s**	6.7 ± 0.1	6.7 ± 0.1	6.7 ± 0.2*	6.3 ± 0.1	6.2 ± 0.2	6.2 ± 0.1	<0.0001

Table S3
Blood differentials and CRP levels in male and female healthy volunteers at baseline and post cantharidin-induced blister harvest. Data are shown as mean ± SD with statistical significance determined using using two-way ANOVA with post-hoc Sidak's tests (note there were no significant differences between the sexes or on the effects of cantharidin on circulating markers or within sex).

		Hb (g/dL)	Platelet count ($x10^9/L$)	WBC ($x10^{11}/L$)	Neutrophils ($x10^9/L$)	Lymphocytes ($x10^9/L$)	Monocytes ($x10^9/L$)	CRP (mg/L)
**Male**	**Baseline**	15.0 ± 0.3	234.7 ± 15.2	5.4 ± 0.3	3.0 ± 0.3	1.8 ± 0.1	0.4 ± 0.04	<5
	**Post-blister**	15.0 ± 0.3	242.2 ± 12.9	5.6 ± 0.4	3.2 ± 0.3	1.8 ± 0.1	0.4 ± 0.04	<5
**Female**	**Baseline**	13.1 ± 0.3	258.0 ± 12.0	6.3 ± 0.5	3.8 ± 0.4	1.9 ± 0.1	0.4 ± 0.02	<5
	**Post-blister**	12.7 ± 0.2	259.9 ± 12.2	6.0 ± 0.3	3.3 ± 0.2	2.0 ± 0.1	0.4 ± 0.02	<5
	**Sex Comparison**	<0.0001	0.123	0.109	0.133	0.133	0.859	0

Table S4 Cytokine/chemokine array of 24 hour cantharidin-induced blister supernatants in male and female healthy volunteers. Data are shown as mean ± SD with statistical significance determined using using two-way ANOVA with post-hoc Sidak's tests shown as ** for P <0.01 for comparison of time-points between the sexes and no significant differences for within group comparisons to baseline.

		IL-6 (pg/ml)	IL-10 (pg/ml)	CCL-5 (pg/ml)	CXCL-1 (pg/ml)	IL-8 (pg/ml)	MCP-1 (pg/ml)
**Male**	**24 h**	4361 ± 1182	6.6 ± 2.3	11.5 ± 1.8	1046 ± 606.6	5716 ± 1453	6139 ± 2141
	**72 h**	3250 ± 1178	48.1 ± 26.7	38.5 ± 26.0	224.5 ± 36.0	3675 ± 563.8	224.4 ± 26.86
**Female**	**24 h**	5087 ± 1697	18.0 ± 7.3	15.5 ± 2.7	4224 ± 1809	13383 ± 2771**	9658 ± 3113
	**72 h**	5240 ± 3007	30.6 ± 0.0	10.7 ± 0	380.4 ± 124.3	5511 ± 9376	302.6 ± 93.2
	**Sex comparison**	0.452	0.653	0.375	0.221	0.0484	0.527

Table S5 Blister exudate LM-SPM profiles. Exudates were collected from male (n=13) and female volunteers (n=11) 24h after cantharidin application and supernatants were profiling using LC-MS/MS based profiling. Results are mean ± s.e.m. with statistical significance determined using Student's 2-tailed unpaired t-test. - = below limit, limit ≈ 0.1 pg

			Lipid mediators levels (pg/50µL)						
**DHA bioactive metabolome**	**Q1**	**Q3**	**Female**			**Male**			***p* value**
RvD1	375	215	1.6	±	1.1	1.4	±	0.8	0.317
RvD2	375	215	0.4	±	0.7	0.1	±	0.3	0.120
RvD3	375	147	0.0	±	0.1		-		0.143
RvD4	375	101		-			-		
RvD5	359	199	0.3	±	0.3	0.5	±	0.5	0.176
RvD6	359	159		-			-		
17R-RvD1	375	215	1.8	±	1.7	0.8	±	0.9	0.051
17R-RvD3	375	147	0.0	±	0.1	0.1	±	0.1	0.246
PD1	359	153	**0.3**	**±**	**0.6**		**-**		**0.046**
10S,17S-diHDHA (PDX)	359	153	0.7	±	0.6	0.8	±	0.9	0.463
MaR1	359	250	2.5	±	6.4	0.9	±	1.7	0.195
7S,14S-diHDHA	359	221	0.1	±	0.3	0.3	±	0.7	0.201
17-HDHA	343	245	22.7	±	17.5	242.6	±	589.5	0.115
14-HDHA	343	205	194.2	±	108.2	163.8	±	120.4	0.263
7-HDHA	343	141	2.7	±	1.6	3.2	±	2.7	0.282
4-HDHA	343	101	3.6	±	1.7	4.6	±	4.8	0.264
DHA	327	283	3031.3	±	1309.2	2789.6	±	1334.4	0.33
**EPA bioactive metabolome**									
RvE1	349	161	0.4	±	1.0	0.4	±	0.6	0.403
RvE2	333	199	1.0	±	1.1	0.8	±	1.2	0.344
RvE3	333	245	4.0	±	6.6	1.6	±	3.1	0.125
18-HEPE	317	259	2.1	±	4.8	4.0	±	9.6	0.288
15-HEPE	317	219	7.9	±	11.3	101.7	±	227.7	0.094
12-HEPE	317	179	20.1	±	52.2	27.3	±	70.8	0.390
5-HEPE	317	115	0.4	±	1.2	1.6	±	4.9	0.23.2
EPA	301	257	975.5	±	622.1	1231.0	±	857.7	0.210
**AA bioactive metabolome**									
LXA_4	351	115		-			-		
LXB_4	351	221	5.4	±	3.6	3.5	±	2.0	0.062
5S,15S-diHETE	335	115	15.0	±	19.6	9.9	±	6.6	0.193
AT-LXA_4	351	115	0.2	±	0.3	0.2	±	0.3	0.493
AT-LXB_4	351	221	0.6	±	1.2	0.3	±	0.5	0.198
LTB_4	335	195	**0.6**	**±**	**0.3**	**1.2**	**±**	**1.0**	**0.037**
PGD_2	351	189	**16.2**	**±**	**12.9**	**8.9**	**±**	**4.0**	**0.032**
PGE_2	351	189	228.1	±	205.6	134.9	±	72.2	0.070
$PGF_{2\alpha}$	353	193	1116.6	±	550.0	808.9	±	468.2	0.076
TxB_2	369	169	805.2	±	796.4	426.8	±	559.6	0.093
20-HETE	319	301	15.5	±	11.9	19.9	±	17.8	0.246
15-HETE	319	219	144.8	±	211.0	848.2	±	1605.3	0.082
12-HETE	319	179	551.2	±	223.4	514.5	±	311.8	0.374
5-HETE	319	115	9.3	±	5.3	13.3	±	8.6	0.098
AA	303	259	4320.9	±	1980.6	4714.8	±	2041.8	0.319

Table S6 Lipid mediator profiles in plasma from male or female volunteers following typhoid vaccination Plasma was collected and LM levels were assessed using LM-profiling. Results are expressed as pg/mL; mean ± s.e.m. ; n = 4 volunteers per group. - = below limit, limit ≈ 0.1 pg
.

			Lipid mediators levels (pg/mL)		
**DHA bioactive metabolome**	**Q1**	**Q3**	**Male**	**Female**	***p* value**
RvD1	375	233	0.1 ± 0.0	0.1 ± 0.0	0.280
RvD2	375	141	0.3 ± 0.1	0.4 ± 0.2	0.312
RvD3	375	147	0.2 ± 0.1	0.1 ± 0.0	0.187
RvD4	375	101	0.5 ± 0.5	0.1 ± 0.1	0.230
RvD5	359	199	1.2 ± 0.2	2.0 ± 0.7	0.152
RvD6	359	101	0.3 ± 0.1	0.2 ± 0.1	0.340
17R-RvD1	375	141	0.1 ± 0.0	0.1 ± 0.1	0.312
17R-RvD3	375	147	0.2 ± 0.1	0.1 ± 0.0	0.187
PD1	359	153	0.2 ± 0.0	0.2 ± 0.1	0.296
17R-PD1	359	153	0.2 ± 0.1	0.1 ± 0.1	0.329
22-OH-PD1	375	153	0.4 ± 0.4	0.7 ± 0.7	0.343
10S,17S-diHDHA (PDX)	359	153	0.6 ± 0.0	0.9 ± 0.3	0.098
MaR1	359	221	-	-	
MaR2	359	191	2.9 ± 1.3	2.5 ± 1.0	0.395
22-OH-MaR1	375	221	0.4 ± 0.4	0.1 ± 0.2	0.247
7S,14S-diHDHA	359	221	-	-	0.232
4S,14S-diHDHA	359	101	0.3 ± 0.3	0.1 ± 0.1	0.178
**EPA bioactive metabolome**					
RvE1	349	161	0.2 ± 0.1	0.4 ± 0.1	0.118
RvE2	333	159	0.1 ± 0.1	-	0.369
RvE3	333	201	0.4 ± 0.2	0.9 ± 0.4	0.094
**AA bioactive metabolome**					
LXA_4	351	217	0.1 ± 0.0	0.1 ± 0.0	0.298
LXB_4	351	221	**5.2 ± 2.6**	**-**	**0.028**
5S,15S-diHETE	335	235	4.9 ± 2.6	1.1 ± 0.8	0.082
AT-LXA_4	351	217	0.2 ± 0.2	0.1 ± 0.1	0.317
AT-LXB_4	351	221	1.1 ± 0.6	1.2 ± 0.8	0.440
LTB_4	335	195	**0.6 ± 0.0**	**0.5 ± 0.0**	**0.006**
6-trans- LTB_4	335	195	0.1 ± 0.0	-	0.168
12-epi-6-trans- LTB_4	335	195	0.2 ± 0.1	-	0.099
5S,12S-diHETE	335	195	0.1 ± 0.0	0.1 ± 0.0	0.414
20-OH-LTB_4	351	195	0.1 ± 0.0	-	0.085
PGD_2	351	189	3.5 ± 1.8	4.4 ± 2.6	0.382
PGE_2	351	189	5.7 ± 2.4	6.9 ± 3.7	0.379
$PGF_{2\alpha}$	353	193	3.8 ± 1.0	6.0 ± 3.0	0.220
TxB_2	369	169	97.8 ± 36.3	108.7 ± 52.7	0.426

Supplementary Figures

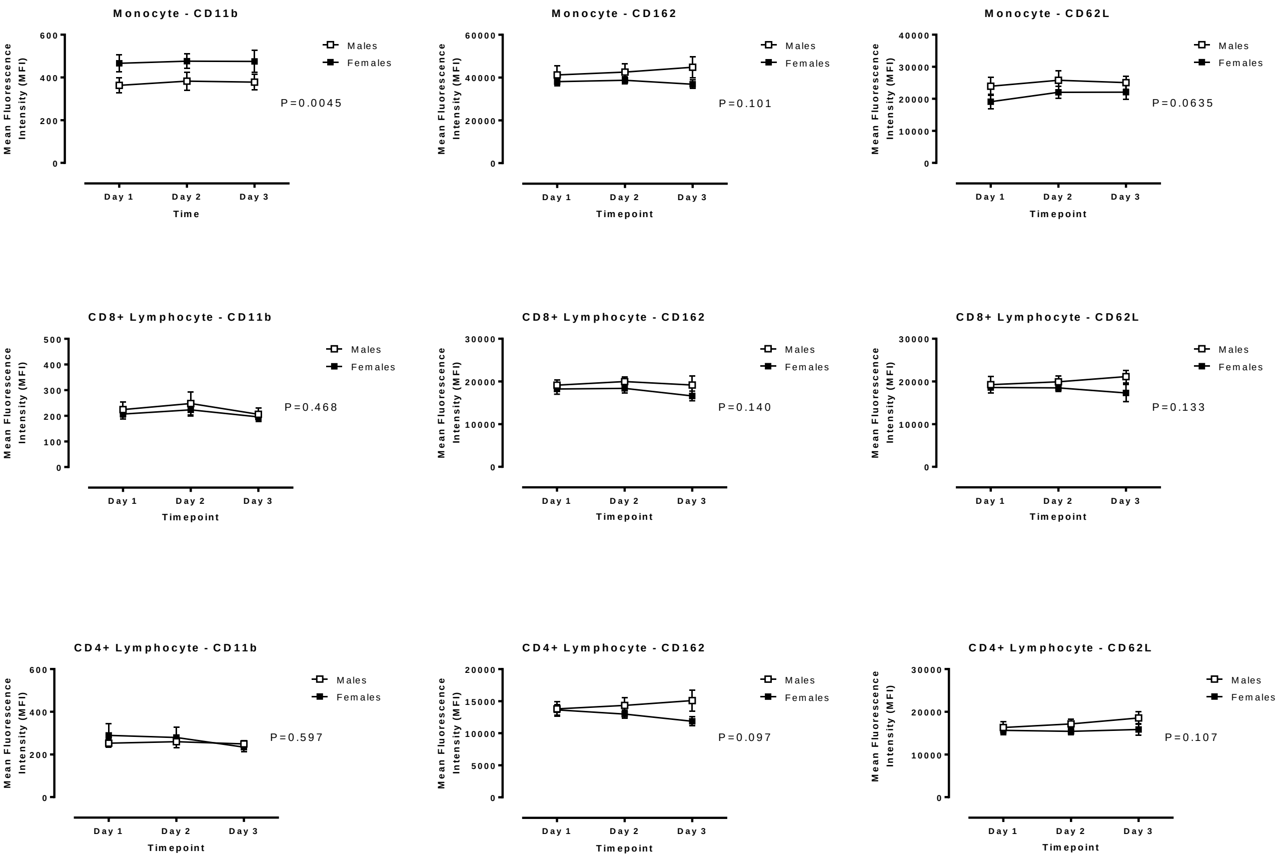

Figure S1 – Changes in expression of CD11b, CD62L and CD162 on monocytes, CD4+ and CD8+ lymphocytes in male and female healthy volunteers measured at baseline, 8 hours and 32 hours following typhoid vaccine. Data expressed as mean ± s.e.mean for n=12 for each sex for all the panels. Statistical significance determined using two-way ANOVA with Sidak's post-hoc analysis for all the panels. MFI = Median fluorescence intensity.

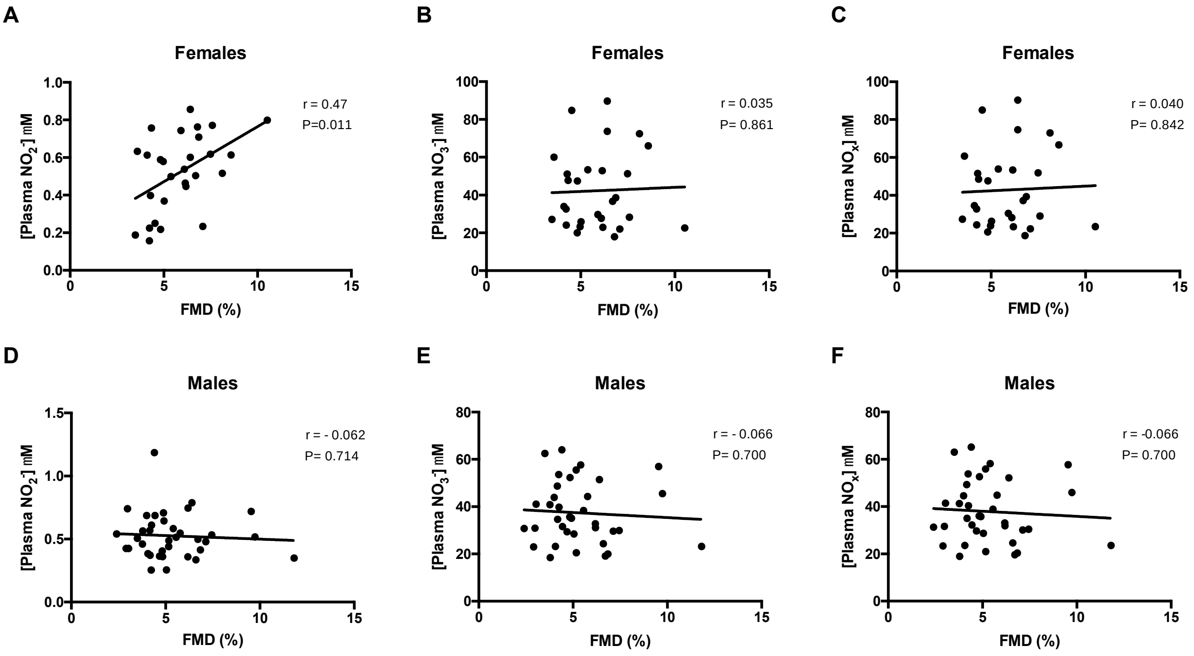

Figure S2 Correlations between FMD responses in healthy volunteers and levels of plasma NO metabolites NO_2^-, NO_3^- and NO_X in females (A-C) and males (D-F). Data from pre-vaccine baseline and 8 and 32 hours post-vaccine are collated. All graphs show Pearson linear regression, and statistical significance of r-value for all the panels.

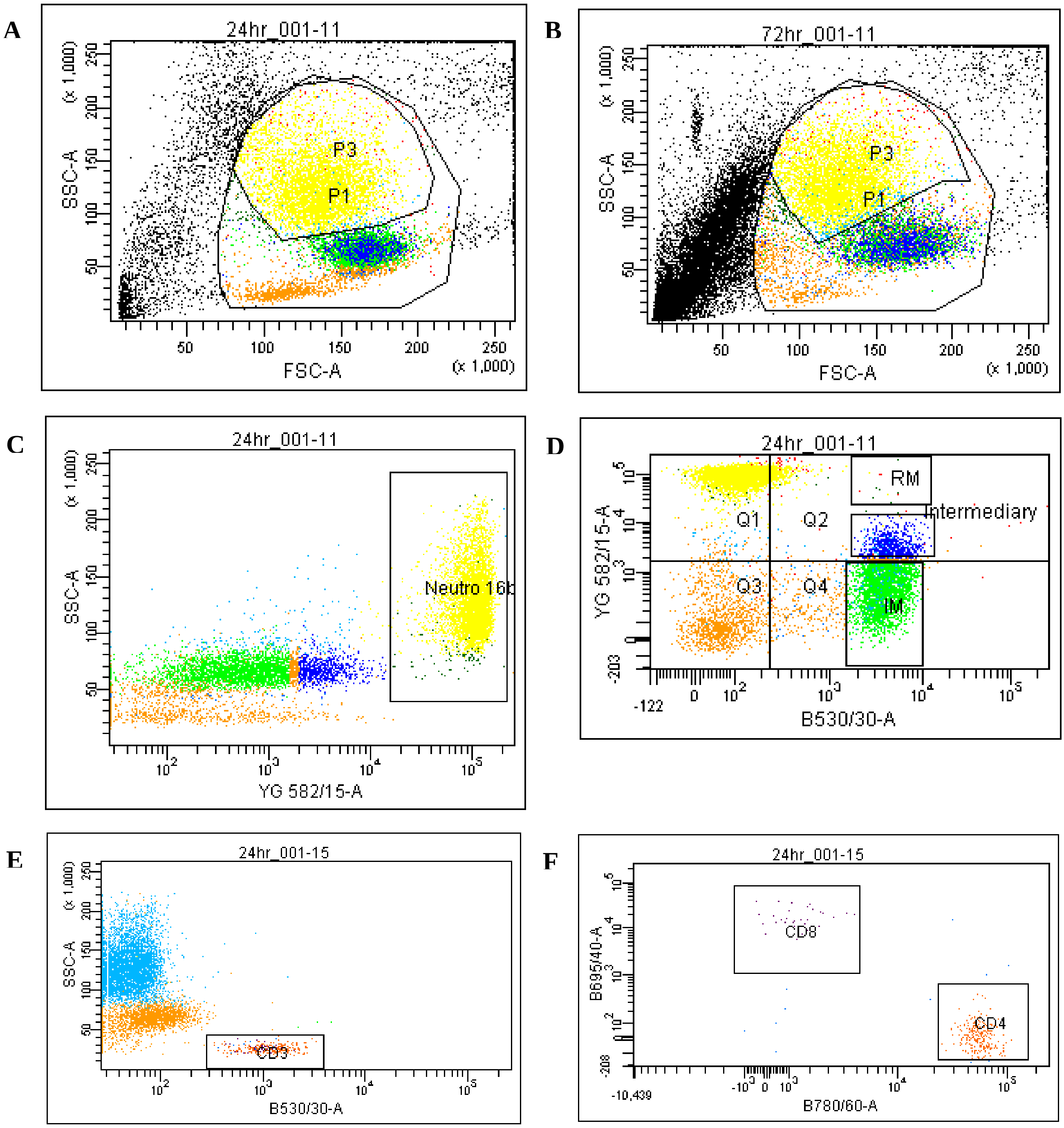

Figure S3 – Representative dot plots to identify leucocyte sub-populations collected in cantharidin induced blister fluid in healthy volunteers: A-B) Characteristic FSC/SSC dot plot from a 24hrs and 72hrs blister fluid respectively; **C)** Density dot plot of neutrophils (yellow-gated cells) confirmed by anti-CD16b+ binding; **D)** Density dot plot of monocytes showing presence of CD14+/CD16- classical inflammatory (IM) monocytes (green-gated), CD14+/CD16+ intermediate monocytes (blue-gated) and a fraction of CD14lo/CD16++ resident (RM) monocytes confirmed by appropriate binding patterns with anti-CD14 and/or anti-CD16; **E)** Density dot plot of lymphocytes (red-gated cells) confirmed by anti-CD3 binding and **F)** Density dot plot showing T-cell sub-types with anti-CD4+ (orange-gated cells) and anti-CD8+ (blue-gated cells) binding.

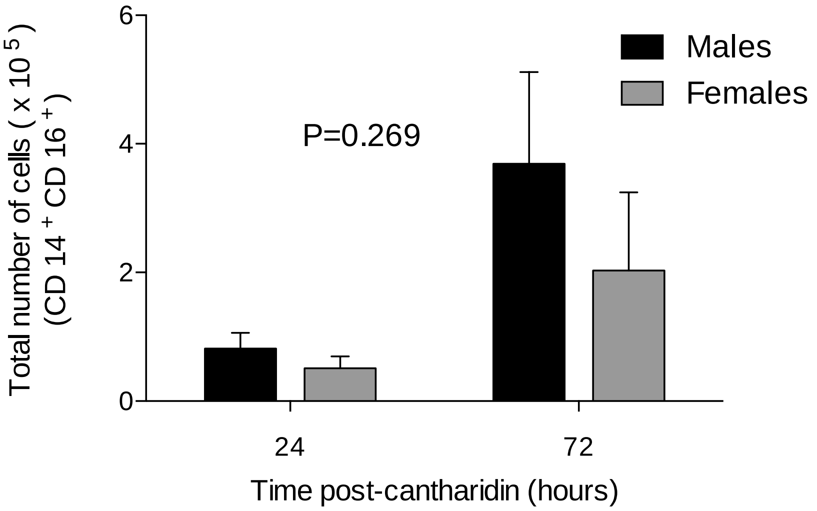

Figure S4 – Intermediate Monocyte count in cantharidin-induced blister fluid at 24 hours and 72 hours post application in healthy volunteers (male n=13-15, female n=14-16). Data shown as mean ± s.e.mean with statistical significance determined using two-way ANOVA to compare between sexes on each day; not significant.

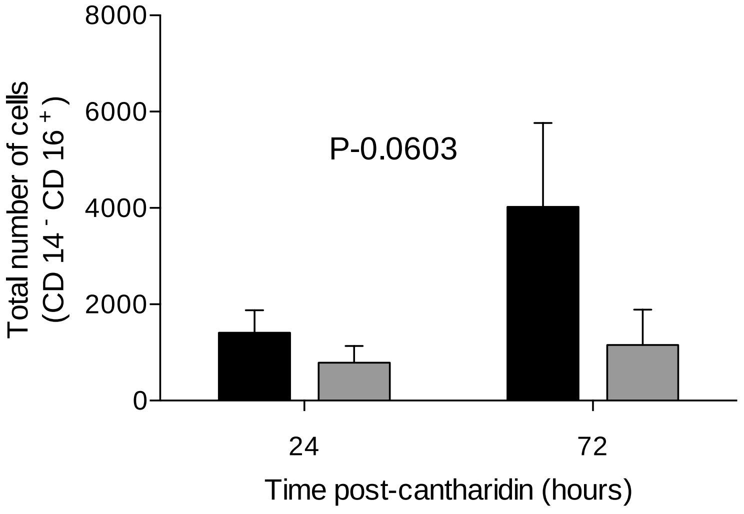

Figure S5 – Resident Monocyte in cantharidin-induced blister fluid at 24 hours and 72 hours post application in healthy volunteers (male n=13-15, female n=14-16). Data shown as mean ± s.e.mean with statistical significance determined using two-way ANOVA to compare between sexes on each day; not significant.

Figure S6. Distinct plasma LM-SPM profiles in males and females following typhoid vaccination. Plasma was collected for healthy volunteers following typhoid vaccine administration and LM levels were assessed using LM-profiling (see methods for details). (A) *left panel* Representative Multiple Reaction Monitoring chromatograms for identified mediators. *a*=Δ6-trans-LTB_4, *b*=12-epi, Δ6-trans-LTB_4. *right panel* MS-MS fragmentation and diagnostic ions employed for LXB_4 and RvE1 identification. m/z, mass-to-charge ratio; M-H, molecular ion. (B) *top panel* 2-dimentional score plot of human plasma LM-SPM profiles. *Bottom panel* 2-dimentional loading plot. Grey ellipse in the score plot denotes 95% confidence regions. Results are representative n=4 male and n=4 female healthy volunteers. (C-D) Cumulative levels for each of the bioactive LM families identified. Results are mean±s.e.m n=4 volunteers per group. * p<0.05 vs Male volunteer plasma values.

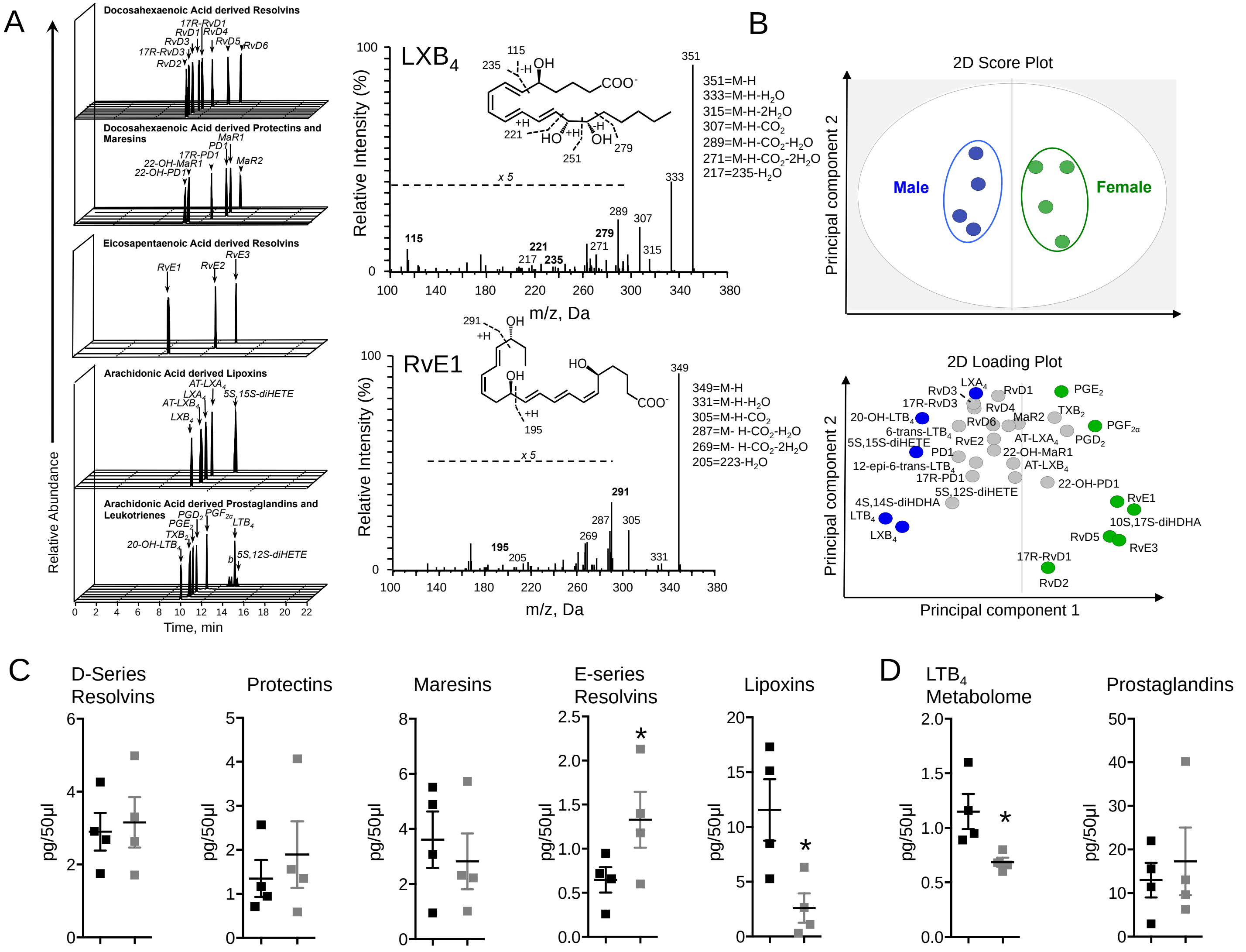

Supplement: Supplemental data [file jci-127-89429-s001.pdf]
